# Universal Mitochondrial Multi-Locus Sequence Analysis (mtMLSA) to Characterise Populations of Unanticipated Plant Pest Biosecurity Detections

**DOI:** 10.3390/biology11050654

**Published:** 2022-04-24

**Authors:** Ela Hiszczynska-Sawicka, Dongmei Li, Karen F. Armstrong

**Affiliations:** 1 AgResearch, Lincoln Science Centre, Christchurch 8140, New Zealand; 2 Plant Health and Environment Laboratory, Diagnostic and Surveillance Services, Ministry for Primary Industries, Auckland 1140, New Zealand; Dongmei.Li@mpi.govt.nz; 3 Bio-Protection Research Centre, Lincoln University, Christchurch 8140, New Zealand; Karen.Armstrong@lincoln.ac.nz

**Keywords:** pest insects, quarantine diagnostics, pest management, PCR primers, DNA sequencing

## Abstract

**Simple Summary:**

Agricultural and environmental sustainability requires effective biosecurity responses that prevent the establishment or spread of exotic insect pests. Understanding where new detections may have come from or if recurrent detections are connected contributes to this. Suitable population genetic markers use relatively rapidly evolving gene regions which render the PCR method species-specific at best. Because resource limitations mean these are pre-emptively developed for the highest risk species, populations of other exotic pests are unable to be characterised at the time. Here we have developed a generic method that is useful across species within the same taxonomic Order, including where there is little or no prior knowledge of their gene sequences. Markers are formed by concomitant sequencing of four gene regions. Sequence concatenation was shown to retrieve higher resolution signatures than standard DNA barcoding. The method is encouragingly universal, as illustrated across species in ten fly and 11 moth superfamilies. Although as-yet untested in a biosecurity situation, this relatively low-tech, off-the-shelf method makes a proactive contribution to the toolbox of quarantine agencies at the time of detection without the need for impromptu species-specific research and development.

**Abstract:**

Biosecurity responses to post-border exotic pest detections are more effective with knowledge of where the species may have originated from or if recurrent detections are connected. Population genetic markers for this are typically species-specific and not available in advance for any but the highest risk species, leaving other less anticipated species difficult to assess at the time. Here, new degenerate PCR primer sets are designed for within the Lepidoptera and Diptera for the 3′ COI, ND3, ND6, and 3′ plus 5′ 16S gene regions. These are shown to be universal at the ordinal level amongst species of 14 and 15 families across 10 and 11 dipteran and lepidopteran superfamilies, respectively. Sequencing the ND3 amplicons as an example of all the loci confirmed detection of population-level variation. This supported finding multiple population haplotypes from the publicly available sequences. Concatenation of the sequences also confirmed that higher population resolution is achieved than for the individual genes. Although as-yet untested in a biosecurity situation, this method is a relatively simple, off-the-shelf means to characterise populations. This makes a proactive contribution to the toolbox of quarantine agencies at the time of detection without the need for unprepared species-specific research and development.

## 1. Introduction

The first post-border detections of an exotic pest can signal the start of an invasion. This is a time when effort and resources spent on eradication or containment might be most effective [1,2]. Knowledge of the pests’ potential association with either offshore populations or prior detections can support this by providing clues to pathways that may need to be curbed [3] or as evidence of recurrent invasions that would impede eradication or containment [4]. The same can also contribute to simulation models aimed at improving risk assessment and pest management [5]. Direct observation alone, or historical clues as to the source or pathway can be ambiguous, misleading, or non-existent, particularly for species that are either highly dispersive [6,7], polyphagous, and thus difficult to link to a host plant pathway, or arrive as hitchhikers via unpredictable routes. Indirect evidence using molecular markers to associate or characterise a population can provide valuable supplementary information or may even be the only avenue available [8]. Here we aim to address a perceived gap for unanticipated arrivals or genetically poorly understood pest species with the proposal of a generic method that can be applied in the short time frame of a quarantine response.

Suitable molecular tools involve PCR amplicon sequencing and sequence haplotype matching of gene regions that are variable enough to encompass appropriate population-level diversity [3]. PCR priming sites are, therefore, at best, only conserved enough for very closely related species. Consequently, the effort and resources to develop the method by species have been constrained to those pests considered very high-risk based on their economic, environmental, or social impact and global invasion patterns [9,10,11,12,13,14]. Unfortunately, a lot of exotic insects which arrive, and those which may become established, do not have this status but represent potential hazards nonetheless, for which population-level data would be beneficial [15]. They might not be considered serious pests elsewhere or not have been intercepted previously at the ports of entry [16], so contingencies are not prioritised. Such species are also usually poorly documented genetically, if at all. An example is the number of economically damaging species first recorded in New Zealand between 1960 and 1975 [17]; of 32 listed, 21 remain (as of September 2020) with <100 DNA sequence records in GenBank, and of which a third have none. Protocols and choice of gene targets would therefore need to be determined at the time of detection. This is impractical given the narrow time frames available to improve the odds of a successful response and gathering such data risks being dismissed as an option. For all such circumstances, the best prospect would be access to generic population genetic methods that can be deployed immediately across a wide taxonomic range, as has been enabled for species identification [18,19].

Genetic approaches to the population for ecological, epidemiological, and pest management applications often capitalise on the relatively high evolutionary rate of certain nuclear gene regions. Nuclear microsatellite loci have been preferred as markers of geographic origin for high impact plant pests [3,20,21,22], but their design requires species-specific priming sites and genomic locations. Even with improvements in technology [23] and greater availability of genome sequences [24] that make finding such loci easier, testing them to confirm suitable population-level heterogeneity is extremely time-consuming. They are also not trivial to use because of a complex evolution [25,26] and the potential for cross-amplification of non-orthologous loci [27]. Single nucleotide polymorphisms (SNPs) in moderately evolving nuclear coding and non-coding regions can be more robust for population structure and assignment [28] and have conserved regions for PCR priming [29]. They have also been used to assess colonisation history and recent pest invasions [9]. However, they can be equally difficult to develop beyond a particular target species, and there are numerous molecular, theoretical and practical challenges for population assignment, such as recombination, heterozygosity, insertion and deletion polymorphisms, low divergence, and difficult sequence alignment [30,31]. Overall, rapidly evolving nuclear gene regions that are useful for studying evolutionary processes are unrealistic for biosecurity incursions of a few individuals of understudied, unanticipated species.

Mitochondrial DNA (mtDNA) SNP haplotyping, on the other hand, has been favoured for entomological population history and phylogeographic applications for practical reasons, including conserved sequence regions flanking suitably evolving loci for PCR primer design and no intragenic indel or intron sequences or heterozygous bases to complicate alignment. Compared to nuclear gene regions, a large and accumulating volume of insect mtDNA data is also becoming publicly available for use [32,33], including from complete mitogenome sequences [34] that in themselves have been used to assess populations, including phylogeography of an invasive species (Lepidoptera: *Lymantria dispar*) [35] and diversity within species across a large section of the Diptera [36]. Although various issues with reliability as a marker of population diversity have been raised [37,38], mtDNA is still recognised as the cheapest way to provide initial data on otherwise uncharacterised species [37] and is preferred over nuclear markers for recent geographic history [39]. However, finding one PCR protocol that can be applied across broad taxonomic ranges is challenging.

Here we describe a relatively simple and taxonomically-broad means to generate population markers for unanticipated exotic insect detections. It relied on finding gene regions that provide useful population-level variation as well as flanking sequence stretches conserved enough for between-species PCR priming of suitably-sized amplicons. These form the basis of multilocus sequence analysis (MLSA), as distinct from the allelic focus of multilocus sequence typing (MLST) [40]. MLSA typically concatenates the sequences of a small number of protein-coding genes and is a method typically associated with microbial studies, including for phylogeography and origins [41], that has rarely been applied in entomology [42]. Generally, it appears to be used as species- or at best genus-specific in terms of the diversity of taxa that can be evaluated with the same methodological conditions.

Our study has designed universal primers that now allow the concurrent amplification of six mitochondrial loci, each containing population-level variation, but that can be amplified at the higher taxonomic level amongst families within an order. These loci are the COI 5′ barcode region, which is likely to be used anyway at the time of detection to confirm species identity [43,44], the 3′ COI region, the ND3 and ND6 complete genes, plus the overlapping 5′ and 3′ 16S ribosomal RNA regions that capture the whole gene. These loci were decided upon based on known intra-specific insect variation [45,46,47,48,49], but it was also that important they were represented by an abundance of publicly available data from which reliable universal priming sites could be located. In silico analysis confirmed the synergistic effect of concatenating the sequences of these loci to improve population-level resolution. While empirical testing of the respective primer sets confirmed they are largely successful within Diptera and within Lepidoptera.

## 2. Materials and Methods

### 2.1. Insect Specimens

Specimens from 15 dipteran (n = 62) and 15 lepidopteran (n = 124) families were gathered from both fresh locally collected New Zealand taxa, as well as preserved specimens of species exotic to New Zealand (Tables S1 and S2). A variety of standard field collection methods (sweep net, light trap, pheromone trap, rearing from hand-collected immatures) were used as appropriate for the species being collected. All insect samples were stored in 70% ethanol at −20 °C until DNA extraction. The New Zealand specimens were identified to the most confident taxonomic level (Brian Patrick, http://www.wildlands.co.nz/services/, accessed on 16 November 2014). Exotic species intercepted at the New Zealand border had been identified by the relevant taxonomist in that country or by New Zealand’s Ministry for Primary Industries Plant Health and Environment Laboratory (MPI-PHEL) (Auckland, New Zealand). Although the focus was on families that contain plant pests, the availability of specimens from other families were opportunistically included to improve the taxonomic range used for empirical testing primer universality. All families were represented by at least two specimens wherever possible. Molecular identification (see below) was used for specimens unable to be identified morphologically.

### 2.2. Choice of Loci and Universal PCR Primer Design

The mtDNA primers developed here have been limited to demonstrating universality across only families within the orders Diptera and Lepidoptera. The mtDNA gene regions targeted were based on those being highly represented in GenBank to enable robust primer design and for containing suitable population variability. The latter was determined using the sequences obtained from genome data in GenBank for species represented by several geographic populations. Population variation was assessed as the number of haplotypes amongst the samples used. The final loci were chosen within the COI, ND3, ND6, and 16S genes to be short enough to be sequenced in their entirety but long enough to contain several variable nucleotide sites.

Primer sets targeting COI-3′, ND3, ND6 and two adjacent 16S rRNA gene fragments were either adopted from previous studies, including Diptera and Lepidoptera or designed here. For the latter, up to 5000 GenBank (NCBI, 2013–2015) sequences for each gene plus their neighbouring regions were retrieved (data not included, retrieved between 2013 and 2015). These were compiled as a single alignment for each gene within each Order using Geneious^®^ 7.0.5 software (http://www.geneious.com, accessed on 13 November 2021 [50]) with the built-in Geneious or ClustalW aligner. The most conserved regions within each gene alignment were identified by visual inspection as potential PCR priming sites. Primers of 18–26 nucleotides long were designed manually, including sites with multiple bases for up to 1536× degeneracy to increase universality. Hairpins, self-dimers and primer-dimers were avoided through Geneious^®^ 7.0.5.

### 2.3. Molecular Analyses

Insect specimens unable to be identified to species morphologically were identified from their COI barcode according to the protocol of Hebert et al. [51]. DNA was extracted either from leg muscles or from whole insects using a Genomic DNA tissue kit (Geneaid Biotech Ltd., New Taipei City, Taiwan) and stored at −20 °C pending analysis. Similarly, for specimens intercepted at the New Zealand border, DNA was extracted from a single leg of an adult or segment of a larva using the DNeasy Blood and Tissue kit (Qiagen, Valencia, CA, USA) as per the manufacturer’s instructions. For all specimens, the COI barcode region was PCR amplified using MyTaq^TM^ Polymerase (Bioline, London, UK), 50 °C annealing temperature and the barcode primers, LCO1490: 5′-GGTCAACAAATCATAAAGATATTGG-3′- and HCO2198: 5′-TAAACTTCAGGGTGACCAAAAAATCA-3′- [52]. PCR amplified fragments were sequenced at Macrogen (Seoul, Korea) with the same primers. COI sequences were run through the BOLD Identification System (IDS) for COI using the Species Level Barcode Records Database (October 2016) [33] to call species identity (Table S1).

To test all primer sets and the 28S positive control (below), PCR amplification was performed in 20 μL reactions using 1 μL of DNA extract (of varying concentration), 1 U MyTaq^TM^ HS DNA Polymerase (Bioline, London, UK) or Platinum^®^ Taq DNA Polymerase (Invitrogen, Carlsbad, CA, USA), 3 mM MgCl_2_ and final primer concentrations of 0.4–0.8 μM. Initial denaturation was performed at 95 °C for 5 min, followed by 35 or 40 cycles of 30 s at 94 °C, 30 s annealing from 42 °C to 50 °C (Tables S3 and S4), 30 s at 72 °C, with a final 5 min extension at 72 °C. Amplifications were carried out either in a Mastercycler^®^ thermocycler (Eppendorf, Hamburg, Germany) or an MJ Mini^TM^ personal Thermal Cycler (Bio-Rad, Hercules, CA, USA). Some protocol validation amplifications were also conducted with the quarantine-testing facilities at MPI-PHEL (Auckland, New Zealand) and used a SimpliAmp or Veriti Thermal Cycler (Applied Biosystems, Foster City, CA, USA). The amplicons were resolved by electrophoresis in 2% agarose gels containing RedSafeTM (iNtRON, Sangdaewon-Dong, Korea). PCR success was considered as the visible presence of a single band of the anticipated size. Selected amplicons were sent to Macrogen (Seoul, Korea) for cleanup and sequencing in both directions to confirm that the correct loci were amplified. The presence of multiple bands was not scored as a successful PCR.

An empirical test of haplotype variation, that had previously been indicated by in silico analysis (Section 2.2), was undertaken for ND3 only as an exemplar of all the gene regions. DNA from locally collected lepidopteran species supplied as more than five individuals from one or two geographic locations were used, with PCR and sequence analysis carried out as above and the primer set ND3-J-Gly-Lepido/ND3-N-Arg-Lepido. Where amplicons were sequenced, the same PCR primers were used for sequencing, and all produced high-quality trace files.

### 2.4. Primer Universality

All primer sets (Tables S3 and S4) were initially tested with DNA prepared from *Drosophila melanogaster* (Drosophilidae), *Bactrocera dorsalis*, and *Bactrocera tryoni* (Tephritidae) for the Diptera or *Pieris brassicae* and *Pieris rapae* (Pieridae) for the Lepidoptera. To account for failed PCRs and ensure that DNA quality was not a factor, positive reactions with each DNA sample used the conserved 28S rDNA D2-D3 gene region with primers D2A: 5′-ACAAGTACCGTGAGGGAAAGTAGG-3′ and D3B: 5′-TCGGAAGGAACCAGCTACTA-3′ [53,54]. Samples producing negative reactions here as well as with the target gene primers were excluded from further analysis. Primers that failed to amplify, despite a positive 28S reaction, or produced non-specific products were re-run with the high fidelity Accuzyme^TM^ DNA Polymerase (Bioline, London, UK) to test for improved amplification from 3′-5′ proofreading exonuclease activity. Continued failure with some dipteran primers, however, led to them being discarded from further analysis. Negative reactions with no DNA were included to test for potential contamination. The final sets of primers were tested by PCR amplification as above with multiple taxa within 14 and 15 families across each of the Diptera (Table S1) and Lepidoptera (Table S2), respectively.

## 3. Results

### 3.1. Selection of Target Genes and Primers

#### 3.1.1. Haplotype Analysis to Confirm Population-Level Variation

The COI, ND3, ND6, and 16S genes were identified as useful for broadly universal primer design based on the larger number and taxonomic breadth of sequences available in GenBank to use. Evidence of population-level variability within these genes was then sought prior to the final selection of the loci for primer development. Using sequences from mitochondrial genome datasets in GenBank rather than from single-locus submissions allowed comparisons to be made between loci as well as with their concatenation. The COI and 16S genes were each further divided into two slightly overlapping regions equivalent to the sizes of PCR products that are most feasible to sequence entirely from one template, and with a view to standard Sanger sequencing platforms being the most accessible facility. Each species revealed several population haplotypes for the individual loci, with the majority showing more haplotypes when those sequences were concatenated than were apparent for any of the separate gene regions (Table 1). For example, from 31 *Drosophila yakuba* (Drosophilidae) genomes, the greatest number of haplotypes was 11 for COI-3′, compared to 15 for the concatenated sequences. Similarly, for *Bombyx mori* (Bombycidae), out of 29 genomes, the maximum number of haplotypes for any one locus was five compared to 16 for the concatenated sequences. Most species also showed a greater number of haplotypes for the six concatenated loci than for the COI barcode region alone, which was significantly improved upon in some cases (*D. yakuba*, *B. mori*). Further support for the level of population variation at some of these individual loci is provided in Tables S5 and S6, in addition to this data (Table 1), but using single-locus submissions to GenBank.

#### 3.1.2. Ordinal-Level Universal Primer Design

To capitalize on inter-population sequence variability of the targeted genes, the flanking regions required a degree of nucleotide degeneracy to be useful as universal priming sites. Degeneracy was trialled from a factor of 2 to 1536 but was limited to 216 for final testing (Table 2 and Table 3) as higher levels usually gave either no product or multiple bands. Nevertheless, some highly degenerate primers still performed well with *D. melanogaster* and one at 216 for other dipteran species (Table 2 and Table S7). Degeneracy often needed to be introduced in the third position at the 3′ end of the primer, which in most cases did not impede the efficiency of PCR reactions. Higher degeneracy was generally required for Diptera, reflecting the higher sequence variability in these genes than for the Lepidoptera, at least for the taxa retrieved from GenBank. This was except for two successful dipteran primers within the more conserved tRNA genes flanking ND3 and ND6 that needed no degeneracy. Hairpins, self-dimers, and primer-dimers were usually avoided, but even high scores for some of these did not hinder amplifications.

**Figure 1:**
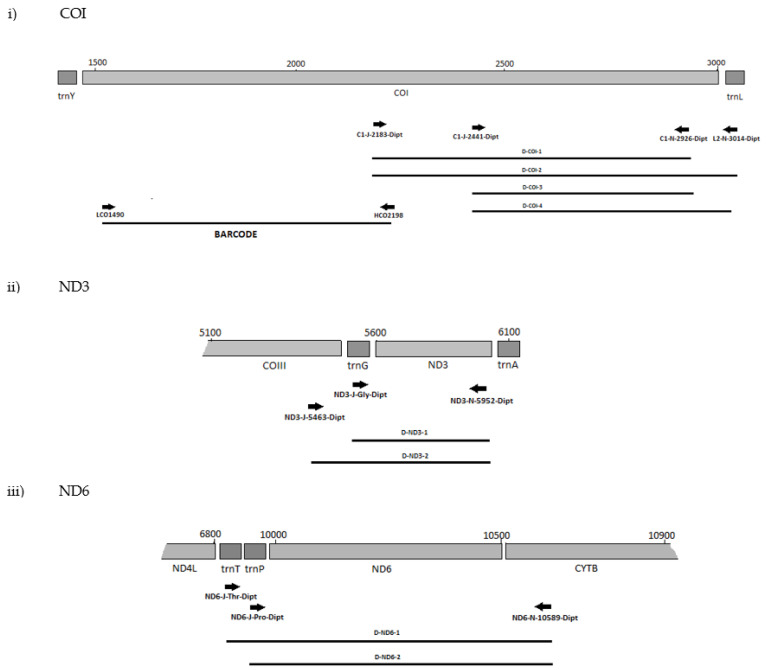

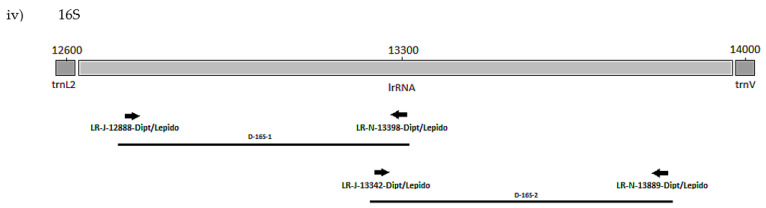
MLSA primer positions for Diptera genes (**i**) COI, (**ii**) ND3, (**iii**) ND6 and (**iv**) 16S.

**Figure 2:**
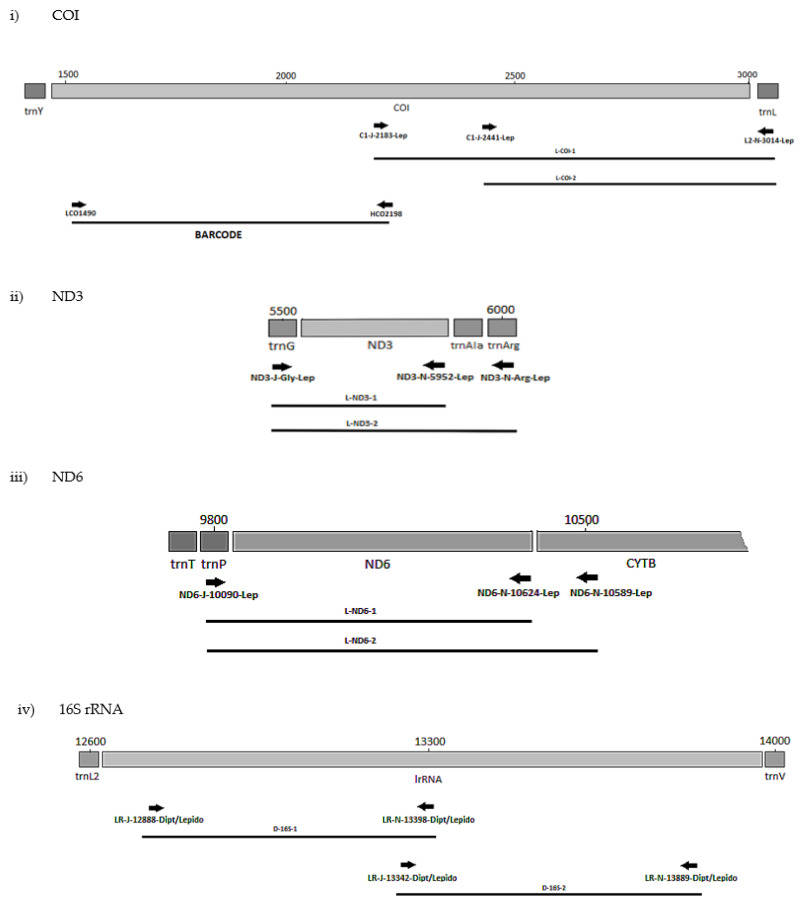
MLSA primer positions for Lepidoptera genes (**i**) COI, (**ii**) ND3, (**iii**) ND6 and (**iv**) 16S.

The positions of the final 10 primer pairings across the four genes (Tables S3 and S4), including the barcode primers, are illustrated in Figure 1 and Figure 2 for the Diptera and Lepidoptera, respectively. For ND6, the reverse primer in CytB was universal across both orders, as were all the primers used to amplify the 16S rRNA gene.

### 3.2. Primer Screening

The range of dipteran and lepidopteran specimens used to test the final primer sets is provided in Tables S1 and S2, respectively, along with their morphological or molecular taxonomic identification and geographic source. Specimens relied on the COI DNA barcode for identification, but for which species level reference sequences were not available, they were identified to family or genus.

#### 3.2.1. Diptera

Of the original 19 Diptera primer pairs proposed (Table S3), preliminary PCR testing with *D. melanogaster* produced fragments of the anticipated size for all but one pair for ND6.

That primer set, ND6-J-10070-Dipt/ND6-N-10589-Dipt/Lepido, also failed with *B. dorsalis* and *B. tryoni*. However, as the reverse primer 10589-Dipt/Lepido was still successful with other forward primers, only the highly degenerate primer ND6-J-10070-Dipt was excluded from further testing. In addition, the primer sets C1-J-2195-Dipt/C1-N-2926-Dipt, C1-J-2195-Dipt/L2-N-3014-Dipt, and C1-J-2441-Dipt/C1-N-2776-Dipt for COI-3′ were unsuccessful with the *Bactrocera* species, and as the position of their amplicons overlapped with the successful sets of D-COI-1 (CI-J-2183-Dipt/CI-N-2926-Dipt) and D-COI-2 (CI-J-2183-Dipt/L2-N-3014-Dipt), they were also excluded from the further testing. All ND3 and 16S primer sets were very efficient with both *D. melanogaster* and all the *Bactrocera* species. Positions of the final primer cohort are illustrated in (Figure 1).

Successful primer sets (Table 3 and Figure 1 and Figure 2) from the preliminary tests were then assessed with a further 37 dipteran taxa across 13 additional families (Table 4). For most of the taxa, all primer sets performed well (Table S7). When considering families represented by more than one specimen, the only exception was the primer sets for both ND genes primers and the fungus gnats of the Sciaroidea, with D-ND3-1, D-ND6-1, and D-ND6-2 negative reactions for the Mycetophilidae and Sciaridae and D-ND3-2 also for the Mycetophilidae. Conclusions at the family level for the Ephydridae with D-ND6-2 are difficult to make with negative reactions for the single representative species *Hydrelia tritici*. The Culicidae and Lonchopteridae were each represented by a single specimen of one species; therefore, while positive reactions can be considered indicative of primer compatibility, conclusions from negative reactions cannot be made (Table S7). The D-ND3-1 generated two products with the lonchopterid specimen, potentially a result of internal priming. Except for the tephritids, none of the families affected here contain plant pest species and had not been included in the primer design process. The few specimens of tephritids that failed also performed poorly with the 5′ COI barcode primers, possibly due to the age of the DNA samples. All these primers performed well with the majority of DNA samples prepared from specimens of exotic species intercepted at New Zealand’s border (Table S7). The main exception was D-ND6-2 which performed inconsistently with the agromyzids. For other species, some samples that gave negative results were positive when re-run with the high-fidelity polymerase Accuzyme (Table 4 and Table S7), possibly due to the 3′-5′ proofreading exonuclease activity overcoming suboptimal template-primer PCR conditions or 3′ primer base mismatches [66]. In summary, these inconsistencies do not undermine the taxonomically broad utility of the Dipteran primer sets.

#### 3.2.2. Lepidoptera

The plant pests *P. brassicae* and *P. rapae* (Pieridae) were used in a preliminary screen of all the lepidopteran primer sets (Table 3, Figure 2). Correct amplicons were produced for all four genes, and so a further 55 species for a total of 15 lepidopteran families were tested. The eight primer sets performed well for most taxa (Table 5). Of the 10 families represented by more than one species, exceptions were for negative reactions for the 5′ 16S set L-16S-2 with the carposinid fruitworm moths and L-ND3-1 with the New Zealand endemic Hepialidae (Table 5 and Table S8). Failures by L-COI-2 for *Weisiana* (Hepialidae) and L-ND6-2 for *Helicoverpa armigera* (Noctuidae) plus several crambid genera did not represent systematic failure at the family level. Although for L-ND6-2, negative reactions by 20 species, including nine of the 10 crambid species and four of the seven geometrid species, suggest that there could be an issue with the primer anchored in the CytB gene, which is the difference with the L-ND6-1 set (Figure 2). Some species failing across a number of primers sets, being *Wiseana umbraculata* (Hepialidae), *Orgyia antiqua* (Erebidae), *Lucinodes cordalis*, *Uresiphyta polygonalis* (Crambidae), and *Grapholita molesta* (Tortricidae), were also represented by single specimens and therefore DNA quality may have been a factor. Otherwise, one primer set for each of the genes (L-COI-1, L-ND3-2, L-16S-1) worked well throughout (Table S8).

DNA barcode identifications for a set of unidentified New Zealand specimens thought to be Lepidoptera revealed that three samples were, in fact, from other insect orders, the Megaloptera (alderflies) and Trichoptera (caddisflies). Interestingly, when tested with the Lepidoptera universal primers, all but L-ND3-1 were successful with the alderfly, *Archichauliodes diversus*, and only L-16S-1 worked for both caddisfly families (Table S8). The novel primer pairs developed here may therefore be universal across a much wider group of insects.

An empirical test of being able to detect population-level variation was tested using ND3 as a representative locus. Sequencing of the amplicons for local species for which several individuals and/or geographic populations were collected confirmed that haplotype variation was detectable (Table 6).

## 4. Discussion

The mtMLSA protocol developed here offers an efficient means of detecting sub-specific genetic variation in dipteran and lepidopteran species, with the emphasis on providing support and options for genetic analysis of understudied pests. For many species, this will allow potentially important information to be obtained at the time of an exotic pest incursion without the need for more time consuming, taxon-specific molecular trials. The key to success was the development of pairs of primers that have a high chance of amplification across many taxa within each order. This was demonstrated for five amplified regions across four mtDNA genes, with eight of the 10 dipteran superfamilies (representing both suborders). Of the two other superfamilies, results for the Culicoidea are inconclusive as it was represented by only one species. The Sciaroidea, represented by two families, was able to be amplified for three of the four genes but failed with both ND6 primer pairs. As one of the two ND3 primer pairs also failed, and both these pairs have a primer in a tRNA region, tRNA position rearrangements could be the cause (see below). Success was also evident with at least one primer set for all four genes across 10 of the 11 lepidopteran superfamilies. The failure of six of the eight primer pairs for the Micropterigoidea was inconclusive as this superfamily was only represented by a single specimen. Lists of taxonomically broad primers are available for various dipteran [67] and lepidopteran [29] loci, as well as across arthropod orders[68]. However, they require numerous primer-pair combinations to be trialled for suitability. Other specific and universal primers pairs are either for mitochondrial regions that are not suited for detecting population variation [69,70] or for preliminary testing, revealing that improvements were necessary for reliable amplification and sequencing within our target orders [46]. Elsewhere, mtMLSA and mtMLST have rarely been used in eukaryotes since their original purpose was for prokaryotes that are devoid of mitochondria. Otherwise, they are apparently designed for single species without the need for taxonomically broad primers [71,72,73].

The development of a usefully generic amplification system is not trivial. The target loci, by necessity, are in more rapidly evolving regions; therefore, the strategy was primarily to adopt a tolerable level of oligonucleotide mismatch without a prohibitively high level of degeneracy. Degeneracy is typically recommended to be less than 128× [74] to minimise both nonspecific amplification for downstream analysis and the number of primer sequence versions in the manufactured product. The latter reduces the concentration of the optimally matching primer as well as interferes with the reaction kinetics at primer locations blocked by poorly matching versions of the primer. Nevertheless, the designs here managed successful PCR and sequencing with degeneracy up to 216×. Although not explored, the effectively low concentration of the optimal primer sequence can be offset by reaction modifications such as touch-down PCR.

In addition to primer sequence, trade-offs need to be made with primer placement. Where both priming sites are within the target gene, such as the COI and 16S loci used here, there is a greater risk of isolating nuclear pseudogene versions of the sequence [75]. Thus, caution should be exercised before assuming the correct protein coding sequence has been amplified [76]. To avoid difficulties with pseudogenes and for easier universal design, anchoring in the more conserved flanking tRNA regions is an option and was employed for some primers. However, with our focus at the ordinal level, primer placement may be vulnerable to tRNA rearrangements [34], which would negate the amplification of the target gene. Within the Diptera, this is known to occur for the midges (Sciaroidea) [77,78]. There, rearrangements include an intercepted transposition of trnN between trnG and ND3, inversions for trnP and trnT, plus trnL is often not present in the standard position according to the *Drosophila* genome [77]. These may explain the apparently consistent difficulty in this study with the Sciaroidea families Mycetophilidae and Sciaridae in their amplification (Table 4) with a trnG-located primer for ND3 and trnP- and trnT-located primers for each of the ND6 sets (Table 2, Figure 1). From a growing literature on lepidopteran mitogenomes, several tRNA rearrangements have also been recorded. These mainly concern differences between the ancient monotrysian moths, to which the Hepialoidea in this study belong, and the much larger common group of the ditrysian moths [79], which contain the vast majority of plant pest species. Nevertheless, none have yet been reported to involve the trnL, trnG, trnA, or trnP regions used here (Table 3). Thus, the poor performance of COI for the hepialids is unlikely to be due to tRNA rearrangement. Usefully, tRNA arrangements have been reported as consistent for several plant pest-containing superfamilies, including the Bombycoidea [80], Gelechioidea [81], Lasiocoidea [82], Noctuoidea [83], Pyraloidea [84], and Tortricoidea [85], as well as the Cossoidea [86] which was not included in the current study. However, differences within a superfamily are possible. For example, in the Zygaenoidea *Histia rhodope* (Zygaenidae) displays a standard ditrysian arrangement [87], but *Parasa consocia* (Limacodidae) shows a trnA translocation [88]. This would challenge the use of the ND3-N-Arg-Lepido primer developed here. An alternative strategy is to place primers in neighbouring protein-coding regions. Simon et al. [68]noted that the only major gene they were unable to develop versatile primers for was ND6, where it was necessary to straddle the region using primers paired between ND4 and CytB. For ND6, we only required a primer in CytB for both orders, but also for ND3 in the Diptera with a primer in COIII.

The value of concatenating individually amplified gene sequences to generate more informative mtMLSA haplotypes was implied in a virtual manner using separate gene sequences from published whole-genome population data but was consistent with that shown empirically elsewhere [72]. However, as the number of loci needed to characterise a population is inversely related to the level of polymorphism offered by those loci [9], and hence to the level of population resolution possible [8], the six gene regions in the current study could still be improved. Certainly, the ability to increase the number of loci sequenced is being constantly improved by technological developments, such as high throughput mito-metagenomic sequencing [82,89] and scanning whole genomes of otherwise poorly characterised species for thousands of SNPs [90]. Such approaches have successfully been applied to various population genetic research questions concerning invasive species [35,91,92]. However, the need for substantial computational power, time, and bioinformatics expertise would more commonly obviate those methods as fit-for-purpose in time- and resource-bound biosecurity responses. The simplicity and universality of the mtMLSA PCR method offsets these constraints, albeit at the potential expense of less distinctive population resolution. Improvements could be achieved by single template multiplex PCR and capitalising on new sequencing innovations to sequence the mixed amplicons directly, for example, the platforms by Oxford Nanopore Technologies (ONT, Oxford, UK) [93]. However, critical to that will still be knowledge of suitable primers. The developments proposed in this study offer such options, including annealing temperatures common across loci as a useful starting point.

Interpreting and understanding the limits of population genetic information is challenging for invasion events where classical methods to assess population structure do not generally apply to the short, anthropogenic influenced timescales and the inevitable coarse-grained geographic resolution that is possible. However, with such data more easily accessed by MLSA, theories can be developed about origins within a few generations of invasion [94], as is likely to be the case at the time of an initial detection of a pest in a new region. Along with relevant situational information, it can provide ideas about the likelihood of multiple or single entries, same source origins, or flag risk pathways to regulatory agencies. This is very much a case of recognising potential incursion associations and alternative hypotheses about origins, as opposed to an assignment of origin per se. The latter is constrained by the lack of geographic reference information in most circumstances [95], even for highly studied economically important pests such as fruit flies, where the generation of such data has been ongoing for decades [3]. The exception is the COI DNA barcode region [51] which has a rapidly growing database that provides an unparalleled source of population-level genetic information for many insect species [96]. As this is frequently used anyway at the time of an incursion to confirm species identity and biosecurity risk [97,98], that data could automatically contribute to the characterisation of the population haplotype. Beyond that, we do not suggest that dedicated efforts be made to develop population reference datasets for the additional loci. Rather, as in the case of New Zealand post-border detections, such as for fruit fly species ([18], D. Li, unpublished), lymantriid moths [18], the large white butterfly [99], brown marmorated stink bug [100], and the small hive beetle [101], efforts are made to obtain sequences of specimens from potential source locations to generate population genetic clues as to pathways and, importantly, propagule number [102]. These sequences, together with previously unpublished data for the New Zealand detections, which may include multilocus mitochondrial sequences, are stored in in-house databases (D. Li and E. Hiszczynska-Sawicka pers com) specifically for the purpose of preparing for future incursions. They also serve to illustrate that the population information generated is useful beyond first detections, helping to develop management strategies for newly established invasive species in advance of their regional establishment, such as that the fall armyworm (Spodoptera frugiperda, Lepidoptera: Noctuidae) [103].

## 5. Conclusions

The use of genetic measures founded on population structure to make policy-level decisions will always be difficult [94,104,105]. However, the ability to contribute at least some population-level knowledge towards preparedness and informed responses is made more practicable here with a method that is technologically realistic for today’s quarantine agencies. Beyond this simple approach, however, the primers will also be useful for state-of-the-art technologies such as Nanopores MinION [93], which is already being taken up in other areas of biosecurity diagnostics [106]. Potentially these priming sites could also be used as a starting point to expedite the extension of the approach to other key plant-pest containing orders, such as Coleoptera and Hemiptera. Inevitably though, new risks to plant health will continue to emerge through changing climate, agricultural practices and trade pathways, and the ability to make population distinctions for unexpected arrivals will remain important [104].

## Figures and Tables

**Table 1:** Haplotypes observed in-silico at six genes from Diptera and Lepidoptera mitochondrial genome population data available in GenBank, with the bold emphasising difference between the haplotypes achieved with COI barcode and concatenated loci.

				Haplotypes (n) and Sequence Length (bp) Per Locus							
				COI Barcode	COI-3’	ND3	ND6	16S-5′ 16S-3′		Concatenated	
Family	Species	Genome Sequence Sources, n	Geographic Populations, n	658 bp	733 bp	387 bp	690 bp	510 bp	550 bp	3528 bp	Ref.
**Diptera**											
Calliphoridae	*Lucilia cuprina*	11	2	**3**	3	2	2	2	3	**4**	[55]
	*Chrysomya megacephala*	46	4	**3**	2	4	2	2	3	**8**	[36]
Drosophilidae	*Drosophila santomea*	17	17	**4**	3	2	2	3	1	**4**	[56]
	*Drosophila yakuba*	31	31	**4**	11	5	8	2	3	**15**	[56]
	*Drosophila simulans*	24	9	**4**	4	4	5	5	3	**7**	[56]
	*Drosophila melanogaster*	13		**4**	4	5	4	1	4	**8**	[57]
Tephritidae	*Bactrocera oleae*	21	10	**13**	10	8	11	5	4	**18**	-
**Lepidoptera**											
Bombycidae	*Bombyx mori*	29	14	**2**	4	5	2	4	4	**16**	[58]
Geometridae	*Biston panterinaria*	10	8	**9**	8	8	6	8	7	**9**	[59]
Papillionidae	*Teinopalpus aureus*	9	5	**3**	4	4	4	2	5	**4**	-

**Table 2:** All primer sequences for Diptera, either adapted from the literature or de-novo designed based on sequences available in GenBank ^1^. Primers selected for final testing are marked (*).

Gene	Primer ^2^	Sequence ^3^	Gene Location ^4^	Degeneracy ^5^	Ref ^6^
3′ COI	C1-J-2183-Dipt (F)	CAACA**Y**TTATT**Y**TG**R**TT**Y**TT**Y**GG	COI	32	[60]
	C1-N-2926-Dipt (R)	CATTC**R**AT**W**GA**W**GA**R**TTTA**R**TTG	COI	32	a
	C1-N-2776-Dipt (R) *	GG**R**TA**R**TC**N**GA**R**TA**H**CG**N**CGNGG	COI	1536	[61]
	C1-N-2944-Dipt (R) *	GG**N**GG**N**GT**R**TTTTG**R**TA**Y**C**Y**TTC	COI	256	a
	L2-N-3014-Dipt (R)	T**Y**CAATGCACTA**D**TCTGCCA**H**A**H**TA	trnL	54	[60]
	C1-J-2195-Dipt (F) *	TTG**R**TT**Y**TT**Y**GG**D**CA**Y**CC**H**GA**R**GT	COI	288	[62]
	C1-J-2441-Dipt (F)	AT**Y**AA**R**ATTTT**Y**AG**H**TGA**H**T**D**GC	COI	216	[60]
ND3	ND3-J-Gly-Dipt (F)	TATATTTGACTTCCAATC	trnG	0	a
	ND3-N-5952-Dipt (R)	TAATAT**N**CCTTG**R**TTTCATTC	ND3	8	[60]
	ND3-J-5463-Dipt (F)	GAAGC**H**GC**H**GC**H**TGATA**Y**TGAC	COIII	54	[63]
ND6	ND6-J-Thr-Dipt (F)	TAAAAACATTGGTCTTG	trnT	0	[64]
	ND6-N-10589-Dipt/Lepido (R)	TAA**W**GA**N**CC**R**AA**R**TTTCATC	CytB	32	a
	ND6-J-Pro-Dipt (F)	TCATTAATC**Y**CCAAA**R**TTA	trnP	4	[65]
	ND6-J-10070-Dipt (F) *	GGA**N**TAAT**NY**T**WY**T**WR**T**H**CAAAC	ND6	1536	a
16S 5′	LR-J-12888-Dipt/Lepido (F)	CCGGTT**B**GAACTCARATCA**Y**GTA	16S	12	[60]
	LR-N-13398-Dipt/Lepido (R)	C**Y**CCT**Y**TTTA**W**CAAAA**M**CAT	16S	16	[60]
16S 3′	LR-J-13342-Dipt/Lepido (F)	CCTTTGCACRGT**Y**A**RR**ATACYGC	16S	32	[60]
	LR-N-13889-Dipt/Lepido (R)	ATTTAT**H**GTACCTTK**K**GTATCAG	16S	12	[60]

^1^ See Table S1 for taxonomic scope of GenBank sequences included; ^2^ Primer pairings and their set names given in Table S3; ^3^ Degenerate bases introduced in this study are underlined and bolded; ^4^ The gene region within which the priming site is located; ^5^ Degeneracy reports the number of all unique sequence combinations that the primer contains; ^6^ Source of original published primers prior to any modification; a = designed in this study.

**Table 3:** All primer sequences for Lepidoptera, either adapted from the literature or de-novo designed based on sequences available in GenBank ^1^.

Gene	Primer ^2^	Sequence ^3^	Gene Location ^4^	Degeneracy ^5^	Ref ^6^
3′ COI	C1-J-2183-Lepido (F)	CAACA**Y**TTATT**Y**TGATT**Y**TT**Y**GG	COI	16	[60]
	L2-N-3014-Lepido (R)	TCCATTACAT**R**TA**D**TCTG**Y**CA**Y**ATTA	trnL	24	[60]
	C1-J-2441-Lepido (F)	ATTAAAATTTT**Y**AG**H**TGA**H**T**R**GC	COI	36	[60]
ND3	ND3-J-Gly-Lepido (F)	AGTATATTT**R**A**Y**TTCCAATC	trnG	4	a
	ND3-N-5952-Lepido (R)	TA**R**TAT**N**TTTTG**R**T**H**TCATTC	ND3	48	[60]
	ND3-N-Arg-Lepido (R)	CTTTTA**D**GTCGAAA**H**TAAATGC	trnA	9	a
ND6	ND6-J-10090-Lepido (F)	ATCWATAATCTCCAAAATTAT	trnP	2	[48]
	ND6-N-10624-Lepido (R)	GGNCCA**W**A**R**AA**R**AT**R**TT**D**GT	ND6	192	[48]
	ND6-N-10589-Dipt/Lepido (R)	TAAWGANCC**R**AA**R**TTTCATC	CytB	32	a
16S 5′	LR-J-12888-Dipt/Lepido (F)	CCGGTT**B**GAACTCARATCA**Y**GTA	16S	12	[60]
	LR-N-13398-Dipt/Lepido (R)	C**Y**CCT**Y**TTTA**W**CAAAA**M**CAT	16S	16	[60]
16S 3′	LR-J-13342-Dipt/Lepido (F)	CCTTTGCACRGT**Y**A**RR**ATACYGC	16S	32	[60]
	LR-N-13889-Dipt/Lepido (R)	ATTTAT**H**GTACCTTK**K**GTATCAG	16S	12	[60]

^1^ See Table S2 for taxonomic scope of GenBank sequences included; ^2^ Primer pairings for final testing and their set names given in Table S4; ^3^ Degenerate bases introduced in this study are underlined and bolded; ^4^ The gene region within which the priming site is located; ^5^ Degeneracy reports the number of all unique sequence combinations that the primer contains; ^6^ Source of original published primers prior to any modification; a = designed in this study.

**Table 4:** Summary of PCR success ^1^ with universal primers ^2^ for individual specimens of Diptera.

Superfamily	Family	Genus ^3^	Species ^3^	N ^4^	D-COI-1	D-COI-2	D-COI-3	D-COI-4	D-ND3-1	D-ND3-2	D-ND6-1	D-ND6-2	D-16S-1	D-16S-2
Chironomoidea	Simuliidae ^5^	*Austrosimulium*	*ungulatum*	2	Y	Y	Y	Y	Y *	Y	Y	Y	Y	Y
Culicoidea	Culicidae ^5^	*-*	*-*	1	Y	Y	N	N	Y	Y	N	N	N	N
Empidoidaea	Dolichopodidae	*-*	*-*	1	Y	Y	Y	Y	Y	Y	Y	Y	Y	Y
	Dolichopodidae	*Ostenia*	*robusta*	1	Y	Y	Y	Y	Y	Y	Y	Y	Y	Y
	Empididae	*-*	*-*	1	Y	Y	Y	Y	N	Y	Y	Y	Y	Y
	Empididae	*-*	*-*	1	Y	Y	Y	Y	Y	Y	Y	Y	Y	Y
	Syrphidae	*Eristalis*	*tenax*	1	Y	Y	Y	Y	Y	Y	Y *	Y	Y	Y
	Syrphidae	*Melangyna*	*novaezealandiae*	2	Y	Y	Y	Y	Y	Y	Y	Y	Y	Y
Ephydroidea	Drosophilidae	*Drosophila*	*melanogaster*	1	Y	Y	Y	Y	Y	Y	Y	Y	Y	Y
	Drosophilidae	*Scuptomyza*	*flava*	1	Y	Y	Y	Y	Y	Y	Y	Y	Y	Y
	Ephydridae	*Hydrelia*	*tritici*	3	Y	Y	Y	Y	Y	Y	Y	N	Y	Y
Oestroidea	Calliphoridae ^5^	*Calliphora*	*stygia*	2	Y	Y	Y	Y	Y	Y	Y	Y	Y	Y
	Calliphoridae ^5^	*-*	*-*	1	Y	Y	Y	Y	Y	Y	Y	Y	Y	Y
	Tachinidae ^5^	*-*	*-*	1	Y	Y	Y	Y	Y	Y	Y	Y	Y	Y
Opomyzoidea	Agromyzidae	*Liriomyza*	*cicerina*	1	Y	Y	Y	Y	Y	Y	Y	Y	Y	Y
	Agromyzidae	*Liriomyza*	*trifoli*	2	Y	Y	Y	Y	Y	Y	Y	N	Y	Y
	Agromyzidae	*Liriomyza*	*-*	2	Y	Y	Y	Y	Y	Y	Y/N	Y/N	Y	Y
	Agromyzidae	*-*	*-*	1	Y	Y	Y	Y	Y	Y	Y	N	N	Y
Platypezoidea	Lonchopteridae	*Leuchoptera*	*bifurcata*	1	Y	Y	Y	Y	Y *	Y	N	Y	Y	Y
Sciaroidea	Mycetophilidae	*-*	*-*	2	Y **	Y/N	Y	Y	N	N	N	N	Y	Y
	Sciaridae	*-*	*-*	1	Y	N	Y	Y	N	Y	N	N	Y	Y
Tephritoidea	Tephritidae	*Anastrepha*	*fraterculus*	1	Y	Y	Y	Y	Y	Y	Y	Y	Y	Y
	Tephritidae	*Anastrepha*	*obliqua*	2	Y	Y	Y	Y	Y	Y	Y	Y	Y	Y
	Tephritidae	*Anastrepha*	*sorocula*	2	Y	Y	Y	Y	Y	Y	Y	Y	Y	Y
	Tephritidae	*Anastrepha*	*zenilda*	2	Y	Y	Y	Y	Y	Y	Y	Y	Y	Y
	Tephritidae	*Bactrocera*	*cucurbitae*	2	Y	Y	Y	Y	Y	Y	Y	Y	Y	Y
	Tephritidae	*Bactrocera*	*dorsalis*	1	N	N	N	Y	Y	Y	Y	Y	Y	Y
	Tephritidae	*Bactrocera*	*facialis*	1	Y	Y	Y	Y	Y	Y	Y	Y	N	Y
	Tephritidae	*Bactrocera*	*jarvisi*	1	Y	Y	Y	Y	Y	Y	Y *	Y *	Y	Y
	Tephritidae	*Bactrocera*	*oleae*	1	Y	Y	Y	Y	Y	Y	Y	Y	Y	Y
	Tephritidae	*Bactrocera*	*psidii*	2	Y ***	Y	Y	Y	Y	Y	Y	Y	Y	Y
	Tephritidae	*Bactrocera*	*tryoni*	1	N	Y	N	Y	Y	Y	Y	Y	Y	Y
	Tephritidae	*Bactrocera*	*tryoni (complex)*	3	Y	Y	Y	Y	Y	Y	Y	Y	Y	Y
	Tephritidae	*Bactrocera*	*xanthodes*	1	N	Y	N	Y	Y	Y	Y	Y	N	Y
	Tephritidae	*Ceratitis*	*capitata*	3	Y	Y	Y	Y	Y	Y	Y	Y	Y	Y
	Tephritidae	*Dacus*	*solominensis*	2	Y	Y	Y	Y	Y	Y	Y	Y	Y	Y
	Tephritidae	*Dirioxa*	*pornia*	3	Y	Y	Y	Y	Y	Y	Y	Y	Y	Y
	Tephritidae	*Rhagoletis*	*completa*	2	Y	Y	Y	Y	Y	Y	Y	Y	Y	Y
	Tephritidae	*Rhagoletis*	*pomonella*	2	Y	Y	Y	Y	Y	Y	Y *	N	Y	Y
None	Stratiomyidae	-	-	1	Y	Y	Y	Y	Y	Y	Y	Y	Y	Y

^1^ Y = positive and N = negative reactions by all specimens for the species (Table S7); * Two products in PCR reaction; ** Positive reaction with Accuzyme; *** Positive reaction with Accuzyme and two products in PCR reaction; ^2^ Primer pair combinations taken from amongst those listed in Table 1; all those for COI are for the 3′ fragment; ^3^ Determined using morphology or DNA barcoding [51]; ^4^ Number of specimens tested; ^5^ Contain pests or parasitoids of animals, not plant

**Table 5:** Summary of PCR success ^1^ with universal primers ^2^ for individual specimens of Lepidoptera.

Superfamily	Family	Genus	Species ^3^	N ^4^	L-COI-1	L-COI-2	L-ND3-1	L-ND3-2	L-ND6-1	L-ND6-2	L-16S-1	L-16S-2
Bombycoidea	Saturniidae	*Argema*	*mittrei*	1	Y	Y	Y	Y	Y	Y	Y	Y
	Saturniidae	*Atherina*	*suraka*	1	Y	Y	Y	Y	Y	Y	N	Y
	Saturniidae	*Graellsia*	*isabellae*	1	Y	Y	Y	Y	Y	Y	Y	Y
Copromorphoidea	Carposinidae	Carposina	-	1	Y	Y	N	Y	Y	Y	Y	N
	Carposinidae	*Coscinoptycha*	*improbana*	3	Y	Y	Y	Y	Y	Y	Y	N
Gelechioidea	Blastobasidae	*Blastobasis*	*tarda*	1	Y	Y	Y	Y	Y	Y	Y	Y
	Oecophoridae	*Leptocroca*	*scholaea*	1	Y	Y	Y	Y	Y	Y	Y	Y
	Oecophoridae	*Barea*	*exacha*	2	Y	Y	Y	Y	Y	N	Y	Y
	Oecophoridae	*Gymnobathra*	*coarctatella*	1	Y	Y	Y	Y	Y	Y	Y	Y
Geometroidea	Geometridae	“*Hydriomena*”	*deltoidata*	1	Y	Y	Y	Y	Y	N	Y	Y
	Geometridae	*Asaphodes*	*chlamydota*	1	Y	Y	Y	Y	Y	Y	Y	Y
	Geometridae	*Chloroclystis*	*filata*	1	Y	Y	Y	Y	Y	N	Y	Y
	Geometridae	*Declana*	*junctilinea*	3	Y	Y	Y	Y	Y	N	Y	Y
	Geometridae	*Epyaxa*	*rosearia*	2	Y	Y	Y	Y	N	Y	Y	Y
	Geometridae	*Poecilasthena*	*schistaria*	7	Y	Y	Y	Y	Y	Y	Y	Y
	Geometridae	*Scopula*	*rubraria*	1	N	Y	Y	Y	Y	N	Y	Y
Hepialoidea	Hepialidae	*Wiseana*	*copularis*	3	Y **	N	N	Y	Y	Y	Y	Y
	Hepialidae	*Wiseana*	*umbraculata*	1	N	N	N	Y	N	N	Y	Y
	Hepialidae	*-*	*-*	1	Y	N	Y	Y	Y	Y	Y	N
Micropterigoidea	Micropterigidae	*Sabatinca*	*aurantissima*	1	N	N	Y	N	N	N	Y	N
Noctuoidea	Erebidae	*Lymantria*	*dispar*	2	Y	Y	Y	Y	Y	Y	Y	Y
	Erebiidae	*Lymantria*	*matura*	2	Y	Y	Y	Y	Y	NT	Y	Y
	Erebiidae	*Lymantria*	*-*	5	Y	Y	Y	Y	Y	Y	Y	Y
	Erebiidae	*Nyctemera*	*annulata*	1	Y	Y	Y	Y	Y	Y	Y	Y
	Erebiidae	*Nyctemera*	*-*	1	Y	Y	Y	Y	Y	N	Y	Y
	Erebiidae	*Orgyia*	*antiqua*	1	N	Y	Ya	Ya	Y	NT	N	N
	Erebiidae	*Orgyia*	*leucostigma*	2	Y	Y	Y	Y	Y	NT	Y	Y
	Erebiidae	*Orgyia*	*pseudotsugata*	2	Y	Y	Y	Y	Y	NT	Y	N
	Erebiidae	*Orgyia*	*thyellina*	2	Y	Y	Y	Y	Y	NT	Y	N
	Erebiidae	*Rhapsa*	*scotosialis*	3	Y	Y	Y	Y	Y	Y	Y	Y
	Noctuidae	*Graphania*	*mutans*	6	Y	Y	Y	Y	Y	Y	Y	Y
	Noctuidae	*Graphania*	*ustistriga*	1	Y	Y	Y	Y	Y	N	Y	Y
	Noctuidae	*Helicoverpa*	*armigera*	7	Y	Y	Y	Y	N	Y	Y	N
	Noctuidae	*Meterana*	*decorata*	1	Y	Y	Y	Y	Y	Y	Y	Y
	Noctuidae	*Proteuxoa*	*comma*	9	Y	Y	Y	Y	Y	Y	Y	Y
	Noctuidae	*Spodoptera*	*litura*	3	Y	Y	Y	Y	Y	Y	Y	Y
	Noctuidae	*Tmetolophota*	*atristriga*	1	Y	Y	Y	Y	Y	Y	Y	Y
Papilionoidea	Lycaenidae	*Lampides*	*boeticus*	1	Y	Y	Y	Y	Y	Y	Y	Y
	Pieridae	*Pieris*	*rapae*	1	Y	Y	Y	Y	Y	Y	Y	Y
	Pieridae	*Pieris*	*brassicae*	2	Y	Y	Y	Y	Y	Y	Y	Y
Pyraloidea	Crambidae	*Crocidolomia*	*pavonana*	1	N	N	N	NT	NT	NT	NT	NT
	Crambidae	*Eudonia*	*octophora*	1	Y	Y	Y	Y	Y	N	Y	Y
	Crambidae	*Eudonia*	*minualis*	3	Y	Y	Y	Y	Y	N	Y	Y
	Crambidae	*Eudonia*	*philerga*	1	Y	Y	Y	Y	N	N	Y	Y
	Crambidae	*Eudonia*	*leptalea*	1	Y	Y	Y	Y	Y	N	Y	Y
	Crambidae	*Eudonia*	*sabulosella*	4	Y	Y	Y	Y	Y	N	Y	Y
	Crambidae	*Glaucocharis*	*auriscriptella*	1	Y	Y	Y	Y	Y	N	Y	Y
	Crambidae	*Lucinodes*	*cordalis*	1	N	N	N	Y	Y	N	Y	N
	Crambidae	*Orocrambus*	*flexuosellus*	3	Y	Y	Y	Y	N	N	Y	Y
	Crambidae	*Scoparia*	*diphtheralis*	1	Y	Y	Y	Y	Y	Y	Y	Y
	Crambidae	*Uresiphyta*	*polygonalis*	1	N	N	N	Y	Y	N	Y	N
	Pyralidae	*Plodia*	*interpunctella*	1	Y	Y	Y	Y	N	N	Y	Y
Tortricoidea	Tortricidae	*Harmologa*	*amplexana*	1	Y	Y	Y	Y	Y	Y	Y	Y
	Tortricidae	*Epiphyas*	*postvittana*	5	Y	Y	Y	Y	Y	NT	Y	Y
	Tortricidae	*Grapholita*	*molesta*	1	N	N	N	Y	Y	Y	N	N
	Tortricidae	*Isotenes*	*miserana*	2	Y	Y	Y	Y	Y	Y	Y	Y
Yponomeutoidea	Plutellidae	*Plutella*	*xylostella*	1	Y	Y	N	Y	Y	N	N	Y

^1^ Y = positive and N = negative reactions by all specimens for the species (Table S7); Ya—reaction weak or very weak, NT—not tested; ** positive with Accuzyme; ^2^ Primer pair combinations taken from amongst those listed in Table 2; all those for COI are for the 3′ fragment; ^3^ Determined using morphology; ^4^ Number of specimens tested.

**Table 6:** Haplotypes identified within locally collected samples.

Family	Species	Sequences (n)	Locations (n)	Sequences in Each Location (n)	Haplotypes (n)			Haplotypes in Each Location (n)	Shared Haplotypes (n)
					ND3	COI	Concatenated		
Crambidae	*Orocrambus flexuoselus*	5	2	3/2	2	-	-	1/2	1
Crambidae	*Eudonia sabulosella*	6	2	3/2	4	-	-	3/2	1
Noctuidae	*Graphania mutants*	5	2	1/4	2	-	-	1/2	1
Noctuidae	*Proteuxoa comma*	11	2	10/1	4	-	-	3/1	0
Geometridae	*Poecilasthena schistaria*	5	1	5	3	4	4	-	-
Geometridae	*Poecilasthena purcharia*	5	1	5	5	5	5	-	-

## Data Availability

Data is contained within the article or supplementary material. All sequences have been submitted to GenBank https://www.ncbi.nlm.nih.gov/genbank/, accessed on 6 April 2022, (see Tables S1 and S2 for accession numbers).

## Supplementary Materials

The following are available online at https://www.mdpi.com/article/10.3390/biology11050654/s1, Table S1: Taxonomic scope of Diptera used for primer design and testing, Table S2: Taxonomic scope of Lepidoptera used for primer design and testing, Table S3: Amplicon length and annealing temperature for each final primer pair: Diptera, Table S4: Amplicon length and annealing temperature for each final primer pair: Lepidoptera, Table S5: Number of haplotypes identified for COI, ND3, ND6, 16S rRNA based on sequences available in GenBank for exemplar Diptera, Table S6: Number of haplotypes identified for COI, ND3, ND6, 16S rRNA based on sequences available in GenBank for exemplar Lepidoptera, Table S7: Primer set PCR success with individual Diptera specimens, Table S8: Primer set PCR success with individual Lepidoptera specimens.
