# Supplementary material for: Universal Mitochondrial Multi-Locus Sequence Analysis (mtMLSA) to Characterise Populations of Unanticipated Plant Pest Biosecurity Detections
Source: Biology (Basel). 2022 Apr 24;11(5):654. doi: 10.3390/biology11050654 (PMC9138331; doi:10.3390/biology11050654)
Supplement: Supplementary file 1 [file biology-11-00654-s001.zip › biology-1657649-supplementary.pdf]

Supplementary Material

Table of Contents:

**Table S1.** Taxonomic scope of Diptera used for primer design and testing	**Page 2**
**Table S2**. Taxonomic scope of Lepidoptera used for primer design and testing	**Page 5**
**Table S3.** Amplicon length and annealing temperature for each final primer pair: Diptera	**Page 9**
**Table S4**. Amplicon length and annealing temperature for each final primer pair: Lepidoptera	**Page 10**
**Table S5**. Number of haplotypes identified in-silico for COI, ND3, ND6, 16S rRNA based on sequences available in GenBank for exemplar Diptera	**Page 11**
**Table S6**. Number of haplotypes identified in-silico for COI, ND3, ND6, 16S rRNA based on sequences available in GenBank for exemplar Lepidoptera	**Page 12**
**Table S7**. Primer set PCR success with individual Diptera specimens	**Page 14**
**Table S8.** Primer set PCR success with individual Lepidoptera specimens	**Page 16**

Table S1. Taxonomic scope of Diptera used for primer design and testing

Superfamily	Family[1]	Genus	Species[2]	Geographic origin [3]			n[4]	GenBank Accession[5]
				Location[3]	Latitude	Longitude		
Anisopodoidea	Anisopodidae [c]							
Asiloidea	Bombylidae [b]							
	Tabanidae [a, c]							
	Therevidae [a]							
Bibionoidea	Bibionidae [b]							
Chironomoidea	Ceratopogonidae [a]							
	Chironomidae [a, c]							
	**Simuliidae** [a, d]	*Austrosimulium*	*ungulatum**	Punakaiki, NZ	-42.092266	171.341055	2	MF141966, 75
Culicoidea	**Culicidae** [a, b, c]	*Unidentified*		Lincoln, NZ	-43.641268	172.475694	1	
Empidoidaea	**Dolichopdidae** [a, b]	*Unidentified*		Lincoln, NZ	-43.646943	172.456843	1	MF141968
		Ostenia	*robusta*	Lincoln, NZ	-43.646943	172.456843	1	MF141958
	**Empididae** [a, b, c]	*Unidentified*		Lincoln, NZ	-43.642968	172.469696	2	MF141961, 69
	**Syrphidae** [a, b, c]	*Eristalis*	*tenax**	Lincoln, NZ	-43.645264	172.463315	1	MF141971
		Melangyna	*novaezealandiae*	Lincoln, NZ	-43.645264	172.463315	2	
Ephydroidea	**Drosophilidae** [a, b, c, d]	*Drosophila*	*melanogaster*	Lab colony, Auckland, NZ	NA	NA	1	
		Scuptomyza	*flava**	Lincoln, NZ	-43.644942	172.464061	1	MF141973
	**Ephydridae** [a, b, c]	*Hydrelia*	*tritici**	Oxford, NZ	-43.223818	172.279836	3	MF141959-60, 67
Lauxanioidea	Lauxaniidae [a, b, c]							
Muscoidea	Scatophagidae[a]							
	Anthomyiidae [a, b, c]							
	Muscidae [a, b, c]							
	Fanidae [a]							
Oestroidea	**Calliphoridae** [a, b, c, d]	*Calliphora*	*stygia**	Lincoln, NZ	-43.644942	172.464061	2	MF141962, 64
		Unidentified		Lincoln, NZ	-43.644942	172.464061	1	
	Oestridae [a, b, c]							
	Rhinophoridae [a]							
	Sarcophagidae [a, b]							

Superfamily	Family[1]	Genus	Species[2]	Geographic origin [3]			n[4]	GenBank Accession[5]
				Location[3]	Latitude	Longitude		
	**Tachinidae** [a, b, c]	*Unidentified*		Lincoln, NZ	-43.644942	172.464061	1	MF141976
Opomyzoidea	**Agromyzidae** [a, b, c, d]	*Liriomyza*	*cicerina**	Unknown	NA	NA	1	MF141965
		Liriomyza	*trifolii*	Ex Fiji‡#	NA	NA	2	MF141914, 19, 28-29, 34
		Liriomyza		Piha, NZ‡	-36.9282	174.45019	2	
		Unidentified		Unknown♯#	NA	NA	1	
Platypezoidea	**Lonchopteridae** [a, c]	*Lonchoptera*	*bifurcata**	Lincoln, NZ	-43.223818	172.279836	1	MF141974
	Phoridae[c]							
Psychodoidea	Psychodidae [a]							
Scatopsidea	Scatopsidae [a, b]							
Sciaroidea	Cecidomyiidae [a, c]							
	Keroplatidae [a, b]							
	**Mycetophilidae** [a]	*Unidentified*		Lincoln, NZ	-43.642968	172.469696	2	MF141970, 72, 51, 54
	Pachyneuridae[c]							
	**Sciaridae** [b]	*Unidentified*		Lincoln, NZ	-43.223818	172.279836	1	
Sciomyzoidea	Conopidae [a]							
Tabanoidea	Tabanidae [a, b]							
Tephritoidea	Sepsidae [a]							
	Piophilidae [c]							
	**Tephritidae** [a, b, c, d]	*Anastrepha*	*fraterculus*	Bahia, Brazil			1	
		Anastrepha	*obliqua*	Texas, USA	NA	NA	1	
				Chiapas, Mexico	14.925806	-92.322195	1	
		Anastrepha	*sorocula*	Londrina, Brazil	NA	NA	1	
				El Araujo, Brazil	14.925806	-92.322195	1	
		Anastrepha	*zenilda*	Mossoró , Brazil	-5.190433	-37.344387	1	
				Rio de Janeiro, Brazil	-22.911014	-43.209373	1	
		Bactrocera	*cucurbitae*	Honiara, Solomon Islands	-9.437797	159.962418	1	
				Bandung, Indonesia	-6.934469	107.604954	1	
		Bactrocera	*dorsalis*	Cook Island	NA	NA	1	MF141955-57
		Bactrocera	*facialis*	Ex Tonga‡#	NA	NA	1	MF141922

Superfamily	Family[1]	Genus	Species[2]	Geographic origin [3]			n[4]	GenBank Accession[5]
				Location[3]	Latitude	Longitude		
		Bactrocera	*jarvisi*	Unknown‡#	NA	NA	1	MF141924
		Bactrocera	*oleae*	Ex Italy‡#	NA	NA	1	
		Bactrocera	*psidii*	New Caledonia	NA	NA	2	MF141952-53
		Bactrocera	*tryoni*	Queensland, Australia	NA	NA		
							1	
		Bactrocera	*tryoni complex*	Auckland, NZ‡##	-36.86667	174.76667	3	MF141911-13; MF141916-19, 25-27, 31-33, 35-37
		Bactrocera	*xanthodes*	Ex Tonga‡#	NA	NA	1	MF141921
		Ceratitis	*capitata*	Hawaii, USA	NA	NA	1	
				Brazil	NA	NA	1	
				Ex Peru‡#	NA	NA	1	
		Dacus	*solominensis*	Guadalcanal, Solomon Islands	NA	NA	2	
		Dirioxa	*pornia*	Australia‡#	NA	NA	3	MF141910, 23
		Rhagoletis	*completa*	Italy	NA	NA	2	
		Rhagoletis	*pomonella*	Illinois, USA	40.11059	-88.20727	2	
Tipulidae	Tipulidae [b, c, d]							
No superfamily	**Stratiomyidae** [a, b]	*Unidentified*		Lincoln, NZ	-43.223818	172.279836	1	MF141963

[1] Families which were also included in alignments for primer design using GenBank data are noted as COI-3’[a], ND3[b], ND6[c], 16S[d]; those included as fresh or preserved specimens for primer testing are noted in **bold**

[2] Where fresh specimens were unable to be identified to species by morphology they were identified by their COI barcode (*)

[3] ‡ provided by the Plant Health & Environment Laboratory, Ministry of Primary Industries, New Zealand; # NZ border intercepts, not from NZ; ## from an incursion response and eradicated, i.e. is not established in NZ

[4] Number of specimens tested

[5] To confirm that the correct genes were amplified, randomly chosen PCR products were sequenced and their GenBank accession numbers given

Table S2. Taxonomic scope of Lepidoptera used for primer design and testing

Superfamily	Family[1]	Genus	Species	Geographic origin[2]			n[3]	GenBank Accession[4]
				Location	Latitude	Longitude		
Alucitoidea	Alucitidae [a, b, c]							
Bombycoidea	Bombycidae [a, b, c, d]							
	**Saturnidae** [a, b, c, d]	*Argema*	*mittrei*	Ex France‡#	NA	NA	1	
		Antherina	*suraka*	Ex UK‡#	NA	NA	1	
		Graellsia	*isabellae*	Ex France‡#	NA	NA	1	
	Sphingidae [a, b, c, d]							
Copromorphoidea	**Carposinidae** [a, b, c, d]	*Carposina*	-	NZ‡ #	NA	NA	1	
		Coscinoptycha	*improbana*	NZ‡	-36.8533606	174.8785093	1	
				NZ‡	-43.6294668	172.7410551	1	
				Unknown‡	NA	NA	1	
Cossoidea	Cossidae [a, b, c]							
Drepanoidea	Drepanidae [a, b, c, d]							
Galacticoidea	Galacticidae [a]							
Gelechioidea	Autostichidae [b, c]							
	Batrachedridae [a]							
	**Blastobasidae**	*Blastobasis*	*tarda*	NZ‡	-36.8119322	175.4757192	1	
	Chimabachidae [a]							
	Cosmopterigidae [a, b, c, d]							
	Depressariidae [a, b, c]							
	Elachistidae [a]							
	Gelechiidae [a, b, c, d]							
	**Oecophoridae** [a, b]	*Barea*	*exarcha*	Lincoln, NZ	-43.644582	172.478232	2	MF142008
		Gymnobathra	*coarctatella*	Lincoln, NZ	-43.644582	172.478232	1	
		Leptocroca	*scholaea*	Lincoln, NZ	-43.644582	172.478232	1	
Geometroidea	**Geometridae** [a, b, c, d]	*Asaphodes*	*chlamydota*	Erwell Forest, NZ	-43.444180	172.306925	1	
		Chloroclystis	*filata*	Lincoln, NZ	-43.644582	172.478232	1	
		Declana	*junctilinea*	Erwell Forest, NZ	-43.444180	172.306925	3	
		Epyaxa	*rosearia*	Lincoln, NZ	-43.644582	172.478232	2	MF142001
		Hydriomena	*deltoidata*	Erwell Forest, NZ	-43.444180	172.306925	1	
		Poecilasthena	*schistaria*	Erwell Forest, NZ	-43.444180	172.306925	7	
		Scopula	*rubraria*	Lincoln, NZ	-43.644582	172.478232	1	

Superfamily	Family[1]	Genus	Species	Geographic origin[2]			n[3]	GenBank Accession[4]
				Location	Latitude	Longitude		
Gracillarioidea	Gracillariidae [a, b, c]							
Hedyloidea	Hedylidae [a]							
Hepialoidea	**Hepialidae** [a, b, c, d]	*Wiseana*	*copularis*	Erwell Forest, NZ	-43.444180	172.306925	3	KY629072-73; KY629075; KY629078-79; MF141938-39
		Wiseana	*umbraculata*	Punakaiki, NZ	-42.092266	171.341055	1	KY629074
		-#	-	NZ#	NA	NA	1	
Hesperioidea	Hesperidae [a, b, c, d]							
Hyblaeoidea	Hyblaeidae [c, d]							
Incurvarioidea	Incurvariidae [a]							
	Prodoxidae [a, c]							
Lasiocampoidea	Lasiocampidae [a, b, c, d]							
Micropterigoidea	**Micropterigidae** [b, c]	*Sabatinca*	*aurantissima*	Canterbury, NZ			1	KY629080
Mimallonoidea	Mimallonidae [c]							
Nepticuloidea	Nepticulidae [c]							
Noctuoidea	**Erebiidae** [a, b, c]	*Lymantria*	*dispar*	Ex Japan‡#	NA	NA	1	MF141906
				West Virginia, USA	39.629681	-79.955944	1	MF142020
		Lymantria	*mathura*	Seoraksan National Park, Korea	38.16691	128.517205	1	MF141904
				Okinawa, Japan	26.696989	127.9735	1	MF142023
		Lymantria	-	Ex Japan‡#	NA	NA	5	MF142026-27
		Nyctemera	*annulata*	Burnham, NZ	-43.608884	172.327582	1	MF141946; 77
		Nyctemera	-	Burnham, NZ	-43.608884	172.327582	1	MF141948
		Orgyia	*antiqua*	Czech Republic	NA	NA	1	MF142017
		Orgyia	*leucostigma*	Ontario, Canada	NA	NA	2	MF141902-03; MF142015-16
		Orgyia	*pseudotsugata*	Oregon, USA	41.901122	-120.438514	1	MF142018
				Idaho, USA	47.33518	-116.888509	1	MF142019
		Orgyia	*thyellina*	Rotorua, NZ (lab colony)	-38.162687	176.256574	2	MF141905; MF142021-22
		Rhapsa	*scotosialis*	Erwell Forest, NZ	-43.444180	172.306925	3	MF141987-89
	**Noctuidae** [a, b, c, d]	*Graphania*	*mutans*	Erwell Forest, NZ	-43.444180	172.306925	1	MF141943; MF142002
				Lincoln, NZ	-43.644582	172.478232	5	MF159105; MF141944-45, 47; MF142003-07;

Superfamily	Family[1]	Genus	Species	Geographic origin[2]			n[3]	GenBank Accession[4]
				Location	Latitude	Longitude		
		Ichneutica	*ustistriga*	Erwell Forest, NZ	-43.444180	172.306925	1	
		Ichneutica	*atristriga*	Erwell Forest, NZ	-43.444180	172.306925	1	KY629071; MF159104; MF141949, 78; MF159104
		Helicoverpa	*armigera*	Christchurch, NZ	-43.530955	172.636646	2	
				Toowoomba, Australia	-27.561019	151.953351	2	
				Wagga wagga, Australia	-35.115	147.367778	2	
				Ex Philippines‡#	NA	NA	1	
		Meterana	*decorata*	Lincoln, NZ	-43.644582	172.478232	1	
		Proteuxoa	*comma*	Erwell Forest, NZ	-43.644582	172.478232	8	MF141942; MF141979-86
				Lincoln, NZ	-43.444180	172.306925	1	
		Spodoptera	*litura*	NZ	-36.8448263	174.7542803	2	
				Ex China‡#	NA	NA	1	
	Notodontidae [a, b, c, d]							
	Thaumetopoeidae [a, b, c, d]							
Papilionoidea								
	**Lycaenidae** [a, b, c, d]	*Lampides*	*boeticus*	Ex Zimbabwe‡#	NA	NA	1	
	Nymphalidae [a, b, c, d]							
	Papilionidae [a, b, c, d]							
	**Pieridae** [a, b, c, d]	*Pieris*	*brassicae*	Nelson, NZ	-41.274518	173.262390	1	
				Kwidzyn, Poland	53.729047	19.021187	1	MF141907; MF141940; MF142009; MF142024
		Pieris	*rapae*	Lincoln, NZ	-43.644582	172.478232	1	MF141908; MF141941; MF142025
	Riodinidae [a, b, c, d]							
Pterophoroidea	Pterophoridae [a, b, c]							
Pyraloidea	**Crambidae** [a, b, c, d]	*Crocidolomia*	*pavonana*	Ex Vanuatu‡#	NA	NA	1	
		Eudonia	*leptalea*	Lincoln, NZ	-43.644582	172.478232	1	
		Eudonia	*minualis*	Erwell Forest, NZ	-43.444180	172.306925	3	
		Eudonia	*octophora*	Erwell Forest, NZ	-43.444180	172.306925	1	
		Eudonia	*philerga*	Lincoln, NZ	-43.644582	172.478232	1	
		Eudonia	*sabulosella*	Erwell Forest, NZ	-43.444180	172.306925	4	MF141990-95
		Glaucocharis	*auriscriptella*	Erwell Forest, NZ	-43.444180	172.306925	1	
		Leucinodes	*cordalis*	NZ‡	-36.7730549	174.7631147	1	
		Orocrambus	*flexuosellus*	Erwell Forest, NZ	-43.641268	172.475694	3	MF141996 - MF142000
		Scoparia	*diphtheralis*	Lincoln, NZ	-43.644582	172.478232	1	KY629076
		Scoparia#	-	Lincoln, NZ	-43.644582	172.478232	7	

Superfamily	Family[1]	Genus	Species	Geographic origin[2]			n[3]	GenBank Accession[4]
				Location	Latitude	Longitude		
		Uresiphita	*polygonalis*	NZ‡#	NA	NA	1	
		-	-	NZ	NA	NA	1	
	**Pyralidae** [a, b, c, d]	*Plodia*	*interpunctella*	Ex Australia‡#	NA	NA	1	
Sesioidea	Castniidae [a]							
	Sesiidae [a, b, c, d]							
Tineoidea	Tineidae [a, c, d]							
Tischerioidea	Tischeridae [b, c]							
Torticoidea	**Tortricidae** [a, b, c, d]	*Epiphyas*	*postvittana*	NZ	NA	NA	5	MF142010-14
		Grapholita	*molesta*	Unknown‡#	NA	NA	1	
		Harmologa	*amplexana*	Lincoln, NZ	-43.644582	172.478232	1	
		Isotenes	*miserana*	Netherlands	-36.85472	174.83167	1	
				NZ‡	NA	NA	1	
		Unidentified		NZ	NA	NA	3	MF141950; MF142028-31
Thyridoidea	Thyrididae [a, c, d]							
Yponeumetoidea	Yponomeutidae [a, c, d]							
	**Plutellidae** [a, b, c, d]	*Plutella*	*xylostella*	Motuakara, NZ	-43.744496	172.587049	1	KY629077
	Lyonetiidae [a, b, c]							
Zygaenoidea	Heterogynidae [d]							
	Lacturidae [d]							
	Somabrachyidae [d]							
	Zygaenidae [a, c, d]							

[1] Families which were also included in alignments for primer design using GenBank data are noted as COI[a], ND3[b], ND6[c], 16S[d]; those included as fresh or preserved specimens for primer testing are noted in **bold**

[2] ‡ Preserved specimens provided by the Plant Health & Environment Laboratory, Ministry of Primary Industries, New Zealand; # NZ border intercepts, not from NZ

[3] Number of specimens tested

[4] To confirm that the correct genes were amplified, randomly chosen PCR products were sequenced and their GenBank accession numbers given

Table S3. Amplicon length and annealing temperature for each primer pair: Diptera

Target gene	Primer pair[1]	Primer pair short name	Length (bp)	Annealing (°C)
**3′ COI**	C1-J-2183-Dipt / C1-N-2926-Dipt *	D-COI-1	743	50
	C1-J-2183-Dipt / C1-N-2776-Dipt		596	42
	C1-J-2183-Dipt / C1-N-2944-Dipt		761	42
	C1-J-2183-Dipt / L2-N-3014-Dipt *	D-COI-2	831	42
	C1-J-2195-Dipt / C1-N-2926-Dipt		731	50
	C1-J-2195-Dipt / L2-N-3014-Dipt		819	50
	C1-J-2195-Dipt / C1-N-2776-Dipt		581	42
	C1-J-2195-Dipt / C1-N-2944-Dipt		749	42
	C1-J-2441-Dipt / C1-N-2926-Dipt *	D-COI-3	485	50
	C1-J-2441-Dipt / L2-N-3014-Dipt *		573	50
	C1-J-2441-Dipt / C1-N-2776-Dipt		384	42
	C1-J-2441-Dipt / C1-N-2944-Dipt	D-COI-4	503	42
**ND3**	ND3-J-Gly-Dipt / ND3-N-5952-Dipt*	D-ND3-1	386	50
	ND3-J-5463-Dipt / ND3-N-5952-Dipt*	D-ND3-2	489	50
**ND6**	ND6-J-Thr-Dipt / ND6-N-10589-Dipt/lepido*	D-ND6-1	555	50
	ND6-J-Pro-Dipt / ND6-N-10589-Dipt/Lepido*	D-ND6-2	693	50
	ND6-J-10070-Dipt / ND6-N-10589-Dipt/Lepido		560	50
**16S rRNA**	LR-J-12888-Dipt/Lepido / LR-N-13398-Dipt/Lepido*	D-16S-1	510	50
	LR-J-13342-Dipt/lepido / LR-N-13889-Dipt/Lepido*	D-16S-2	547	50

[1] Primer sets used for final specimen testing*; primers with high degeneracy (Table 1) are underlined

Table S4. Amplicon length and annealing temperature for each final primer pair: Lepidoptera

Target gene	Primer pair[1]	Primer pair short name	Length (bp)	Annealing (°C)
**COI**	C1-J-2183-Lepido / L2-N-3014-Lepido	L-COI-1	875	50
	C1-J-2441-Lepido / L2-N-3014-Lepido	L-COI-2	617	50
**ND3**	ND3-J-Gly-Lepido / ND3-N-5952-E-Lepido	L-ND3-1	386	48
	ND3-J-Gly-Lepido / ND3-N-Arg-Lepido	L-ND3-2	503	48
**ND6**	ND6-J-10090-Lepido / <u>ND6-N-10624-Lepido</u>	L-ND6-1	530	50
	ND6-J-10090-Lepido / ND6-N-10589-Dipt/Lepido	L-ND6-2	690	50
**16S rRNA**	LR-J-12888-Dipt/Lepido / LR-N-13398-Dipt/Lepido	L-16S-1	510	50
	LR-J-13342-Dipt/Lepido / LR-N-13889-Dipt/Lepido	L-16S-2	547	50

[1] Primer with high degeneracy (Table 1) is underlined

Table S5. Number of haplotypes identified in-silico for COI, ND3, ND6, 16S rRNA based on sequences available in GenBank for exemplar Diptera

Family	Genus	Species	sequences (n)	geographic populations (n)	No of haplotypes							References	PopSet No
					COI barcode 658 bp	COI - 3' 733 bp	ND3 387 bp	ND6 690 bp	16S 510 bp	16S 550 bp	Concatenated[1]		
Calliphoridae	*Lucilia*	*cuprina**	11	2	3	3	2	2	2	3	4	Nelson et al. 2012	
	Chrysomya	*megacephala**	46	4	3	2	4	2	2	3	8	Junquera et al. 2016	
Chironomidae	*Echinocladius*	*martini*	10	6						8		Krosch et al. 2009	
	Echinocladius	*martini*	96	9	51							Krosch et al. 2009	
Drosophilidae	*Drosophila*	*santomea**	17	17	4	3	2	2	3	1	4	Llopart et al. 2014	
	Drosophila	*yakuba**	31	31	4	11	5	8	2	3	15	Llopart et al. 2014	
	Drosophila	*simulans**	24	9	4	4	4	5	5	3	7	Llopart et al. 2014	
	Drosophila	*melanogaster**	13	unknown	4	4	5	4	1	4	8	Wolff et al. 2016	
Muscidae	*Stomoxys*	*calcitrans*	80	20		26						Dsouli-Aymes et al. 2011	313578045
	Stomoxys	*calcitrans*	76	16	62							Marquez et al. 2007	110226793
Mycetophilidae	*Boletina*	*gripha*	4	unknown	4				3			- #	
Sarcophagidae	*Sarcophaga*	*albiceps*	5	5					2			-	327388241
	Sarcophaga	*albiceps*	6	6	4							-	327388241
	Sarcophaga	*peregrina*	9	8					4			-	327388241
	Sarcophaga	*peregrina*	12	9	4							-	327388241
	Sarcophaga	*dux*	7	6					3			-	
	Sarcophaga	*dux*	8	7	2							-	
Sciaridae	*Leptosciarella*	*dentata*	3	unknown					3			-	
Simulidae	*Simulium*	*mie*	3	unknown					3			-	
	Simulium	*venustum*	5	unknown					5			-	
Tephritidae	*Ceratitis*	*capitata*	114	25	27							Barr 2009	
	Bactrocera	*oleae**	21	10	13	10	8	11	5	4	18	-	
	Bactrocera	*cucurbitae*	18	unknown				11				-	
	Ceratitis	*fasciventis*	20	unknown				18				-	
	Ceratitis	*rosa*	10	unknown				10				-	
	Ceratitis	*anonae*	15	unknown				12				-	
	Anastrepha	*obliqua*	37	unknown				37				-	

[1]Concatenated sequences for all four genes based on full mitochondrial genomes available

*whole genome data; # - means unpublished

Table S6. Number of haplotypes identified in-silico for COI, ND3, ND6, 16S rRNA based on sequences available in GenBank for exemplar Lepidoptera

Family	Genus	Species	sequences (n)	geographic populations[2] (n)	No of haplotypes							References	PopSet No
					COI barcode	COI 3'	ND3	ND6	16S		Concatenated[1]		
					658bp	733bp	503bp	690bp	510bp	550bp			
Batrachedridae	*Batrachedra*	*pinicolella*	16	unknown	2							-	
	Batrachedra	*praeangusta*	18	unknown	4							-	
Bombycidae	*Bombyx*	*mori**	29	unknown	2	4	5	2	4	4	16	Li et al. 2010	291575480
Carposinidae	*Carposina*	*sasaki*	104	unknown	48							-[#]	
Castniidae	*Telchin*	*licus*	46	5					14			Silva-Brandao et al. 2013	428621672
Cosmopterigidae	*Cosmopterix*	*moutisella*	9	unknown	2							Telfer et al. 2015	
	Cosmopterix	*teligera*	12	unknown	5							Telfer et al. 2015	
	Cosmopterix	*pulchrimella*	15	unknown	3							Telfer et al. 2015	
Crambidae	*Diatraea*	*saccharalis*	23	5	2							Joyce et al. 2016	
	Diatraea	*saccharalis*	30	unknown	4	8						Silva-Brandao et al. 2015	
	Maruca	*vitrata*	64	unknown	64							Periasamy et al. 2015	767591480
Drepanidae	*Drepana*	*africana*	16	unknown					7			-	1024884107
Erebidae	*Alabama*	*argillacea*	18	unknown				3				-	354801533
	Lymantria	*dispar asiatica*	24	3				6				Wu et al. 2015	756785935
	Lymantria	*dispar dispar*	35	9				7				Wu et al. 2015	756785935
	Lymantria	*dispar japonica*	8	1				1				Wu et al. 2015	756785935
Gelechidae	*Chionodes*	*lugubrella*	10	unknown	10							Mutanen et al. 2012	
	Caryocolum	*pullatella*	11	unknown	7							Mutanen et al. 2012	
	Prolita	*sexpunctella*	13	unknown	7							Mutanen et al. 2012	
Geometridae	*Biston*	*panterinaria**	10	8	9	8	8	6	8	7	9	Cheng et al. 2017	
	Ectropis	*obliqua*	31	unknown	31							-	375280991
Lycanidae	*Aricia*	*saepiolus*	24	11	24							Bromilow et al. 2011	281484791
Noctuidae	*Spodoptera*	*frugiperda*	16	unknown				8				-	354801817
	Heliotis	*virescens*	99	unknown				16				-	359730001
	Heliotis	*virescens*	22	unknown				3				-	354801689
	Helicoverpa	*armigera*	9	2	9							Arnemann et al. 2016	1029978245
Nymphalidae	*Hermeuptychia*	*atalanta*	21	unknown				4				-	354801775
Papilionidae	*Teinopalpus*	*aureus**	9	5	3	4	4	4	2	5	4	-	
Pieridae	*Catopsilia*	*pomona*	9	unknown					9			-	404256588

	Eurema	*hecabe*	42	unknown		18	-	404256588
Plutellidae	*Plutella*	*xylostella*	41	unknown	33		-	37928762
Thyrididae	*Rhodoneura*	*aurata*	47	7		19	Craft et al. 2010	222540163
Tortricidae	*Cydia*	*succedana*	16	14	5		Paynter et al. 2008	189008661
	Cydia	*pomonella*	11	unknown	11		-	20335034
	Grapholita	*molesta*	754	21	41	13	Wei et al. 2015	530848661
Zygaenidae	*Zygaena*	*angelicae elegans*	45	unknown		14	Von Reumont et al. 2012	117615177
	Zygaena	*angelicae*	72	unknown		34	Von Reumont et al. 2012	117615177
	Zygaena	*transalpina astragali*	32	unknown		9	Von Reumont et al. 2012	117615177
	Zygaena	*transalpina centralis*	14	unknown		4	Von Reumont et al. 2012	117615177
	Zygaena	*transalpina dufayi*	10	unknown		8	Von Reumont et al. 2012	117615177
	Zygaena	*hippocrepidus*	69	unknown		23	Von Reumont et al. 2012	117615177
	Zygaena	*mietosa*	20	unknown		7	Von Reumont et al. 2012	117615177
	Zygaena	*transalpina*	181	unknown		80	Von Reumont et al. 2012	117615177

[1]Concatenated sequences for all four genes based on full mitochondrial genomes available; [2]unknown = number of populations not stated in the reference;

*whole genome data ; # unpublished

Table S7. Primer set PCR success[1] with individual Diptera specimens[2]

Superfamily	Family	Genus	Species	D-COI-1	D-COI-2	D-COI-3	D-COI-4	D-ND3-1	D-ND3-2	D-ND6-1	D-ND6-2	D-16S-1	D-16S-2
				C1-J-2183-Dipt	C1-J-2183-Dipt	C1-J-2441-Dipt	C1-J-2441-Dipt	ND3-J-Gly-Dipt	ND3-J-5463-Dipt	ND6-J-Thr-Dipt	ND6-J-Pro-Dipt	LR-J-12888-Dipt/lepido	LR-J-13342-Dipt/Lepido
				C1-N-2926-Dipt	L2-N-3014-Dipt	C1-N-2926-Dipt	L2-N-3014-Dipt	ND3-N-5952-Dipt	ND3-N-5952-Dipt	ND6-N-10589-Dipt/Lepido	ND6-N-10589-Dipt/Lepido	LR-N-13398-Dipt/Lepido	LR-N-13889-Dipt/Lepido
Stratiomyoidea	Stratiomyidae	-	-	Y	Y	Y	Y	Y	Y	Y	Y	Y	Y
Chironomoidea	Simuliidae	*Austrosimulium*	*ungulatum*	Y	Y	Y	Y	Y *	Y	Y	Y	Y	Y
	Simuliidae	*Austrosimulium*	*ungulatum*	Y	Y	Y	Y	Y *	Y	Y	Y	Y	Y
Culicoidea	Culicidae	-	-	Y	Y	N	N	Y	Y	N	N	N	N
Empidoidaea	Dolichopodidae	-	-	Y	Y	Y	Y	Y	Y	Y	Y	Y	Y
	Dolichopodidae	*Ostenia*	*robusta*	Y	Y	Y	Y	Y	Y	Y	Y	Y	Y
	Empididae	-	-	Y	Y	Y	Y	Y a	Y	Y	Y	Y	Y
	Empididae	-	-	Y	Y	Y	Y	Y	Y	Y	Y	Y	Y
	Syrphidae	*Eristalis*	*tenax*	Y	Y	Y	Y	Y	Y	Y *	Y	Y	Y
	Syrphidae	*Melangyna*	*novaezealandiae*	Y	Y	Y	Y	Y	Y	Y	Y	Y	Y
	Syrphidae	*Melangyna*	*novaezealandiae*	Y	Y	Y	Y	Y	Y	Y	Y	Y	Y
Ephydroidea	Drosophilidae	*Drosophila*	*melanogaster*	Y	Y	Y	Y	Y	Y	Y	Y	Y	Y
	Drosophilidae	*Scuptomyza*	*flava*	Y	Y	Y	Y	Y	Y	Y	Y	Y	Y
	Ephydridae	*Hydrelia*	*tritici*	Y	Y	Y	Y	Y	Y	Y	N	Y	Y
	Ephydridae	*Hydrelia*	*tritici*	Y	Y	Y	Y	Y	Y	Y	N	Y	Y
	Ephydridae	*Hydrelia*	*tritici*	Y	Y	Y	Y	Y	Y	Y	N	Y	Y
Oestroidea	Calliphoridae	-	-	Y	Y	Y	Y	Y	Y	Y	Y	Y	Y
	Calliphoridae	*Calliphora*	*stygia*	Y	Y	Y	Y	Y	Y	Y	Y	Y	Y
	Calliphoridae	*Calliphora*	*stygia*	Y	Y	Y	Y	Y	Y	Y	Y	Y	Y
	Tachinidae	-	-	Y	Y	Y	Y	Y	Y	Y	Y	Y	Y
Opomyzoidea	Agromyzidae	*Liriomyza*	*cicerina*	Y	Y	Y	Y	Y	Y	Y	Y	Y	Y
	Agromyzidae	***Liriomyza***	***trifolii***	Y	Y	Y	Y	Y	Y	Y	N	Y	Y
	Agromyzidae	***Liriomyza***	***trifolii***	Y	Y	Y	Y	Y	Y	Y	N	Y	Y
	Agromyzidae	***Liriomyza***	-	Y	Y	Y	Y	Y	Y	N	N	Y	Y
	Agromyzidae	***Liriomyza***	-	Y	Y	Y	Y	Y	Y	Y	Y	Y	Y
	Agromyzidae	-	-	Y	Y	Y	Y	Y	Y	Y	N	N	Y
Platypezoidea	Lonchopteridae	*Lonchoptera*	*bifurcata*	Y	Y	Y	Y	Y *	Y	N	Y	Y	Y
Sciaroidea	Mycetophilidae	-	-	Y	Y	Y	Y	N	N	N	N	Y	Y
	Mycetophilidae	-	-	Y	Y	Y	Y	N	N	N	N	Y	Y
	Sciaridae	-	-	Y	N	Y	Y	N	Y	N	N	Y	Y
Tephritoidea	Tephritidae	*Anastrepha*	*fraterculus*	Y	Y	Y	Y	Y	Y	Y	Y	Y	Y
	Tephritidae	*Anastrepha*	*obliqua*	Y	Y	Y	Y	Y	Y	Y	Y	Y	Y
	Tephritidae	*Anastrepha*	*obliqua*	Y	Y	Y	Y	Y	Y	Y	Y	Y	Y
	Tephritidae	*Anastrepha*	*sorocula*	Y	Y	Y	Y	Y	Y	Y	Y	Y	Y
	Tephritidae	*Anastrepha*	*sorocula*	Y	Y	Y	Y	Y	Y	Y	Y	Y	Y
	Tephritidae	*Anastrepha*	*zenilda*	Y	Y	Y	Y	Y	Y	Y	Y	Y	Y

Tephritidae	*Anastrepha*	*zenilda*	Y	Y	Y	Y	Y	Y	Y	Y a	Y	Y
Tephritidae	*Bactrocera*	*cucurbitae*	Y	Y	Y	Y	Y	Y	Y	Y	Y	Y
Tephritidae	*Bactrocera*	*cucurbitae*	Y	Y	Y	Y	Y	Y	Y	Y	Y	Y
Tephritidae	*Bactrocera*	*dorsalis*	N	N	N	Y	Y	Y	Y	Y	Y	Y
Tephritidae	***Bactrocera***	***facialis***	Y	Y	Y	Y	Y	Y	Y	Y	N	Y
Tephritidae	***Bactrocera***	***jarvisi***	Y	Y	Y	Y	Y	Y	Y **	Y **	Y	Y
Tephritidae	***Bactrocera***	***oleae***	Y	Y	Y	Y	Y	Y	Y	Y	Y	Y
Tephritidae	*Bactrocera*	*psidii*	Y ***	Y	Y	Y	Y	Y	Y	Y	Y	Y
Tephritidae	*Bactrocera*	*psidii*	Y ***	Y	Y	Y	Y	Y	Y	Y	Y	Y
Tephritidae	*Bactrocera*	*tryoni*	Y	Y	N	Y	Y	Y	Y	Y	Y	Y
Tephritidae	***Bactrocera***	***tryoni (complex)***	Y	Y	Y	Y	Y	Y	Y	Y	Y	Y
Tephritidae	***Bactrocera***	***tryoni (complex)***	Y	Y	Y	Y	Y	Y	Y	Y	Y	Y
Tephritidae	***Bactrocera***	***tryoni (complex)***	Y	Y	Y	Y	Y	Y	Y	Y	Y	Y
Tephritidae	***Bactrocera***	***xanthodes***	N	Y	N	Y	Y	Y	Y	Y	N	Y
Tephritidae	***Ceratitis***	***capitata***	Y	Y	Y	Y	Y	Y	Y	Y	Y	Y
Tephritidae	***Ceratitis***	***capitata***	Y	Y	Y	Y	Y	Y	Y	Y	Y	Y
Tephritidae	***Ceratitis***	***capitata***	Y	Y	Y	Y	Y	Y	Y	Y	Y	Y
Tephritidae	*Dacus*	*solominensis*	Y	Y	Y	Y	Y	Y	Y	Y	Y	Y
Tephritidae	*Dacus*	*solominensis*	Y	Y	Y	Y	Y	Y	Y	Y	Y	Y
Tephritidae	***Dirioxa***	***pornia***	Y	Y	Y	Y	Y	Y	Y	Y	Y	Y
Tephritidae	***Dirioxa***	***pornia***	Y	Y	Y	Y	Y	Y	Y	Y	Y	Y
Tephritidae	***Dirioxa***	***pornia***	Y	Y	Y	Y	Y	Y	Y	Y	Y	Y
Tephritidae	*Rhagoletis*	*completa*	Y	Y	Y	Y	Y	Y	Y	Y	Y	Y
Tephritidae	*Rhagoletis*	*completa*	Y	Y	Y	Y	Y	Y	Y	Y	Y	Y
Tephritidae	*Rhagoletis*	*pomonella*	Y	Y	Y	Y	Y	Y	Y ***	N	Y	Y
Tephritidae	*Rhagoletis*	*pomonella*	Y	Y	Y	Y	Y	Y	Y ***	N	Y	Y

[1] Y – positive reaction; N- negative reaction; NT – not tested due to depleted DNA; Ya – reaction weak or very weak; * two products in PCR reaction; ** positive with Accuzyme; *** positive with Accuzyme and two products in PCR reaction

[2] Specimens intercepted at the New Zealand border marked in bold

Table S8. Primer set PCR success[1] with individual Lepidoptera specimens[2]

Superfamily	Family[2]	Genus[2]	Species[2]	L-COI-1	L-COI-2	L-ND3-1	L-ND3-2	L-ND6-1	L-ND6-2	L-16S-1	L-16S-2
				C1-J-2183-Lepido	C1-J-2441-Lepido	ND3-J-GlyLepido	ND3-J-GlyLepido	ND6-J-10090Lep	ND6-J-10090Lep	LR-J-12888-Lepido	LR-J-13342-Lepido
				L2-N-3014-Lepido	L2-N-3014-Lepido	ND3-N-5952-E-Lepido	ND3-N-Arg-Lepido	ND6-N-10624Lep	ND6-N-10589-Lepido	LR-N-13398-Lepido	LR-N-13889-Lepido
Bombycoidea	Saturniidae	***Argema***	***mittrei***	Y	Y	Y	Y	Y	Y	Y	Y
	Saturniidae	***Antherina***	***suraka***	Y	Y	Y	Y	Y	Y	Y	Y
	Saturniidae	***Graellsia***	***isabellae***	Y	Y	Y	Y	Y	Y	Y	Y
Copromorphoidea	Carposinidae	***Carposina***	**-**	Y	Y	N	Y	Y	Y	Y	N
	Carposinidae	***Coscinoptycha***	***improbana***	Y	Y	Y	Y	Y	Y	Y	N
	Carposinidae	***Coscinoptycha***	***improbana***	Y	Y	Y	Y	Y	Y	Y	N
	Carposinidae	***Coscinoptycha***	***improbana***	Y	Y	Y	Y	Y	Y	Y	N
Gelechioidea	Blastobasidae	***Blastobasis***	***tarda***	Y	Y	Y	Y	Y	Y	Y	Y
	Oecophoridae	*Barea*	*exarcha*	Y	Y	Y	Y	Y	N	Y	Y
	Oecophoridae	*Barea*	*exarcha*	Y	Y	Y	Y	Y	Y	Y	Y
	Oecophoridae	*Gymnobathra*	*coarctatella*	Y	Y	Y	Y	Y	Y	Y	Y
	Oecophoridae	*Leptocroca*	*scholaea*	Y	Y	Y	Y	Y	Y	Y	Y
Geometroidea	Geometridae	*Asaphodes*	*chlamydota*	Y	Y	Y	Y	Y	Y	Y	Y
	Geometridae	*Chloroclystis*	*filata*	Y	Y	Y	Y	Y	N	Y	Y
	Geometridae	*Declana*	*junctilinea*	Y	Y	Y	Y	Y	N	Y	Y
	Geometridae	*Declana*	*junctilinea*	Y	Y	Y	Y	Y	N	Y	Y
	Geometridae	*Declana*	*junctilinea*	Y	Y	Y	Y	Y	N	Y	Y
	Geometridae	*Epyaxa*	*rosearia*	Y	Y	Y	Y	N	Y	Y	Y
	Geometridae	*Epyaxa*	*rosearia*	Y	Y	Y	Y	N	Y	Y	Y
	Geometridae	*"Hydriomena"*	*deltoidata*	Y	Y	Y	Y	Y	N	Y	Y
	Geometridae	*Poecilasthena*	*schistaria*	Y	Y	Y	Y	Y	Y	Y	Y
	Geometridae	*Poecilasthena*	*schistaria*	Y	Y	Y	Y	Y	Y	Y a	Y
	Geometridae	*Poecilasthena*	*schistaria*	Y	Y	Y	Y	Y	Y	Y a	Y
	Geometridae	*Poecilasthena*	*schistaria*	Y	Y	Y	Y	Y	Y	Y	Y
	Geometridae	*Poecilasthena*	*schistaria*	Y	Y	Y	Y	Y	Y	Y	Y
	Geometridae	*Poecilasthena*	*schistaria*	Y	Y	Y	Y	Y	Y	Y	Y
	Geometridae	*Poecilasthena*	*schistaria*	Y	Y	Y	Y	Y	Y	Y	Y
	Geometridae	*Scopula*	*rubraria*	N	Y	Y	Y	Y	N	Y	Y
Hepialoidea	Hepialidae	*Wiseana*	*copularis*	Y**	N	N	Y	Y	Y	Y	Y
	Hepialidae	*Wiseana*	*copularis*	Y**	N	N	Y	Y	Y	Y	Y
	Hepialidae	*Wiseana*	*copularis*	N	N	N	Y	Y	Y	Y	Y
	Hepialidae	*Wiseana*	*umbraculata*	N	N	N	Y	N	N	Y	Y

	Hepialidae #	-	-	N	Y	N	Y	Y	Y	Y	Y
Micropterigoidea	Micropterigidae	*Sabatinca*	*aurantissima*	N	N	Y	N	N	N	Y	N
Noctuidea	Erebidae	*Lymantria*	*dispar*	Y a	Y	Y	Y	Y a	NT	Y	Y
	Erebidae	*Lymantria*	*dispar*	Y	Y	Y	Y	Y	Y	Y	Y
	Erebidae	*Lymantria*	*mathura*	Y	Y	Y	Y	Y	NT	Y	Y
	Erebidae	*Lymantria*	*mathura*	Y	Y	Y	Y	Y a	NT	Y	Y
	Erebidae	*Lymantria*	-	Y	Y	Y	Y	Y	NT	Y	Y
	Erebidae	*Lymantria*	-	Y	Y	Y	Y	Y	NT	Y	Y
	Erebidae	*Lymantria*	-	Y	Y	Y	Y	Y	Y	Y	Y
	Erebidae	*Lymantria*	-	Y	Y	Y	Y	Y	Y	Y	Y
	Erebidae	*Lymantria*	-	Y	Y	Y	Y	Y	Y	Y	Y
	Erebiidae	*Nyctemera*	*annulata*	Y	Y	Y	Y	Y	Y	Y	Y
	Erebidae	*Nyctemera*	-	Y	Y	Y	Y	Y	N	Y	Y
	Erebidae	*Orgyia*	*antiqua*	N	Y	Y a	Y a	Y	NT	N	N
	Erebidae	*Orgyia*	*leucostigma*	Y	Y	Y	Y	Y	NT	Y	Y
	Erebidae	*Orgyia*	*leucostigma*	Y	Y	Y	Y a	Y	NT	Y	Y a
	Erebidae	*Orgyia*	*pseudotsugata*	Y a	Y	Y	Y	Y	NT	Y	N
	Erebidae	*Orgyia*	*pseudotsugata*	Y	Y	Y	Y	Y	NT	Y	N
	Erebidae	*Orgyia*	*thyellina*	Y	Y	Y	Y	Y	NT	Y	N
	Erebidae	*Orgyia*	*thyellina*	Y	Y	Y	Y	Y	NT	Y	N
	Erebiidae	*Rhapsa*	*scotosialis*	Y	Y	Y	Y	Y	Y	Y	Y
	Erebiidae	*Rhapsa*	*scotosialis*	Y	Y	Y	Y	Y	Y	Y	Y
	Erebiidae	*Rhapsa*	*scotosialis*	Y	Y	Y	Y	Y	Y	Y	Y
	Noctuidae	*Graphania*	*mutans*	Y	Y	Y	Y	Y	Y	Y	Y
	Noctuidae	*Graphania*	*mutans*	Y	Y	Y	Y	Y	Y	Y	Y
	Noctuidae	*Graphania*	*mutans*	Y	Y	Y	Y	Y	Y	Y	Y
	Noctuidae	*Graphania*	*mutans*	Y	Y	Y	Y	Y	Y a	Y	Y
	Noctuidae	*Graphania*	*mutans*	Y	Y	Y	Y	Y	Y	Y	Y
	Noctuidae	*Graphania*	*mutans*	Y	Y	Y	Y	Y	Y	Y	Y
	Noctuidae	*Ichneutica*	*ustistriga*	Y	Y	Y	Y	Y	N	Y	Y
	Noctuidae	*Helicoverpa*	*armigera*	Y a	Y	Y	Y	N	NT	Y	Y a
	Noctuidae	*Helicoverpa*	*armigera*	Y	Y	Y	Y	N	NT	Y	Y a
	Noctuidae	*Helicoverpa*	*armigera*	Y a	Y	Y a	Y	N	NT	Y	Y a
	Noctuidae	*Helicoverpa*	*armigera*	Y a	Y	Y a	Y	N	NT	Y	Y a
	Noctuidae	*Helicoverpa*	*armigera*	Y a	Y	Y	Y	N	NT	Y	Y a
	Noctuidae	*Helicoverpa*	*armigera*	Y	Y	Y	Y	N	NT	Y	Y a
	Noctuidae	*Helicoverpa*	*armigera*	Y	Y	Y	Y	N	Y	Y	Y a
	Noctuidae	*Meterana*	*decorata*	Y	Y	Y	Y	Y	N	Y	Y
	Noctuidae	*Proteuxoa*	*comma*	Y	Y	Y	Y	Y	Y	N	Y

	Noctuidae	*Proteuxoa*	*comma*	Y	Y	Y	Y	Y	Y	Y	Y
	Noctuidae	*Proteuxoa*	*comma*	Y	Y	Y	Y	Y	Y	Y	Y
	Noctuidae	*Proteuxoa*	*comma*	Y	Y	Y	Y	Ya	Y	Y	Y
	Noctuidae	*Proteuxoa*	*comma*	Y	Y	Y	Y	Y	Y	Y	Y
	Noctuidae	*Proteuxoa*	*comma*	Y	Y	Y	Y	Y a	Y	Y a	Y
	Noctuidae	*Proteuxoa*	*comma*	Y	Y	Y	Y	N	Y	Y	Y
	Noctuidae	*Proteuxoa*	*comma*	Y	Y	Y	Y	N	Y	Y	Y
	Noctuidae	*Proteuxoa*	*comma*	Ya	Y	Y	Y	Y	N	Y	Y
	Noctuidae	***Spodoptera***	***litura***	Y	Y	Y	Y	Y	Y	Y	Y
	Noctuidae	*Spodoptera*	*litura*	Y	Y	Y	Y	Y	Y	Y	Y
	Noctuidae	*Spodoptera*	*litura*	Y	Y	Y	Y	Y	Y	Y	Y
	Noctuidae	*Ichneutica*	*atristriga*	Y	Y	Y	Y	Y	Y	Y	Y
Papilionoidea	Lycaenidae	***Lampides***	***boeticus***	Y	Y	Y	Y	Y	Y	Y	Y
	Pieridae	*Pieris*	*brassicae*	Y	Y	Y	Y	Y	Y	Y	Y
	Pieridae	*Pieris*	*brassicae*	Y	Y	Y	Y	Y	Y	Y	Y
	Pieridae	*Pieris*	*rapae*	Y	Y	Y	Y	Y	Y	Y	Y
Pyraloidea	Crambidae	*Crocidolomia*	*pavonana*	N	N	N	NT	NT	NT	NT	NT
	Crambidae	*Eudonia*	*leptalea*	Y	Y	Y	Y	Y	N	Y	Y
	Crambidae	*Eudonia*	*minualis*	Y	Y	Y	Y	Y	N	Y	Y
	Crambidae	*Eudonia*	*minualis*	Y	Y	Y	Y	Y	N	Y	Y
	Crambidae	*Eudonia*	*minualis*	Y	Y	Y	Y	Y	N	Y	Y
	Crambidae	*Eudonia*	*octophora*	Y	Y	Y	Y	Y	N	Y	Y
	Crambidae	Eudonia	philerga	Y	Y	Y	Y	N	N	Y	Y
	Crambidae	*Eudonia*	*sabulosella*	Y	Y	Y	Y	Y	N	Y	Y
	Crambidae	*Eudonia*	*sabulosella*	Y	Y	Y	Y	Y	N	Y	Y
	Crambidae	*Eudonia*	*sabulosella*	Y	Y	Y	Y	Y	N	Y	Y

Superfamily	Family[2]	Genus[2]	Species[2]	L-COI-1	L-COI-2	L-ND3-1	L-ND3-2	L-ND6-1	L-ND6-2	L-16S-1	L-16S-2
				C1-J-2183-Lepido	C1-J-2441-Lepido	ND3-J-GlyLepido	ND3-J-GlyLepido	ND6-J-10090Lep	ND6-J-10090Lep	LR-J-12888-Lepido	LR-J-13342-Lepido
				L2-N-3014-Lepido	L2-N-3014-Lepido	ND3-N-5952-E-Lepido	ND3-N-Arg-Lepido	ND6-N-10624Lep	ND6-N-10589-Lepido	LR-N-13398-Lepido	LR-N-13889-Lepido
	Crambidae	*Eudonia*	*sabulosella*	Y	Y	Y	Y	Y	N	Y	Y
	Crambidae	*Glaucocharis*	*auriscriptella*	Y	Y	Y	Y	Y	N	Y	Y
	Crambidae	***Leucinodes***	***cordalis***	N	Y a	N	Y	Y	N	Y	N
	Crambidae	*Orocrambus*	*flexuosellus*	Y	Y	Y	Y	N	N	Y	Y
	Crambidae	*Orocrambus*	*flexuosellus*	Y	Y	Y	Y	N	N	Y	Y
	Crambidae	*Orocrambus*	*flexuosellus*	Y	Y	Y	Y	N	N	Y	Y
	Crambidae	***Uresiphita***	***polygonalis***	N	Y a	N	Y	Y	N	Y	N
	Crambidae	*Scoparia*	*diphtheralis*	Y	Y	Y	Y	Y	Y	Y	Y
	Crambidae	*Scoparia#*	-	Y	Y	Y	Y	Y	Y	Y	Y
	Crambidae	*Scoparia#*	-	Y	Y	Y a	Y	Y	Y	Y	Y a
	Crambidae	*Scoparia#*	-	Y a	Y	Y	Y	Y	Y	Y	Y
	Crambidae	*Scoparia#*	-	Y	Y	Y	Y	Y	Y	Y	Y
	Crambidae	*Scoparia#*	-	Y	Y	Y	Y	Y	Y	Y	Y
	Crambidae	*Scoparia#*	-	Y	Y	Y	Y	Y	Y	Y	Y
	Crambidae	*Scoparia#*	-	Y	Y	Y	Y	Y	Y	Y	Y
	Crambidae #	-	-	Y	Y	Y	Y	Y	Y	Y	Y
	Pyralidae	***Plodia***	***interpunctella***	Y	Y	Y	Y	N	N	Y	Y
Tortricoidea	Tortricidae	*Epiphyas*	*postvittana*	Y	Y	Y	Y	Y	NT	Y	Y
	Tortricidae	*Epiphyas*	*postvittana*	Y	Y	Y	Y	Y	NT	Y	Y
	Tortricidae	*Epiphyas*	*postvittana*	Y	Y	Y	Y	Y	NT	Y	Y
	Tortricidae	*Epiphyas*	*postvittana*	Y	Y	Y	Y	Y	NT	Y	Y
	Tortricidae	*Epiphyas*	*postvittana*	Y	Y	Y	Y	Y	NT	Y	Y
	Tortricidae	***Grapholita***	***molesta***	N	N	N	Y	Y	Y	N	N
	Tortricidae	*Harmologa*	*amplexana*	Y	Y	Y	Y	Y	Y	Y	Y
	Tortricidae	***Isotenes***	***miserana***	N	Y	Y a	Y	Y	Y	Y	Y
	Tortricidae	***Isotenes***	***miserana***	Y	Y	Y	Y	Y	Y	Y	Y
Yponomeutoidea	Plutellidae	*Plutella*	*xylostella*	Y	Y	N	Y	Y	N	N	Y
						**OTHER ORDERS**					
Order: Megaloptera	Corydalidae	*Archichauliodes*	*diversus#*	Y	Y	N	Y	Y	Y	Y	Y

Order: Trichoptera	Hydropsychidae	*Hydropsyche tepoka#*	N	N	N	N	N	N	Y	N
	Leptoceridae	*Hudsonema amabile#*	N	N	Y	Y	N	Y	Y	N

[1] Y – positive reaction; N- negative reaction; NT – not tested due to depleted DNA; Ya – reaction weak or very weak; * two products in PCR reaction; ** positive with Accuzyme

[2] Specimens intercepted at the New Zealand border marked in bold; # specimens were identified using BOLD and sequence similarity clusters of 92-97% to the family level, 96-97% to the genus level and 99.2-100% to the species level.

References cited

Arnemann, J. A., W. J. James, T. K. Walsh, J. V. C. Guedes, G. Smagghe, E. Castiglioni, and W. T. Tay. 2016. Mitochondrial DNA COI characterization of *Helicoverpa armigera* (Lepidoptera: Noctuidae) from Paraguay and Uruguay. Genet. Mol. Res. 15: 1-8.

Barr, N. B. 2009. Pathway Analysis of *Ceratitis capitata* (Diptera: Tephritidae) using mitochondrial DNA. J. Econ. Entomol. 102: 401-411.

Bromilow, S. M., F. A. H. Sperling. 2011. Phylogeographic signal variation in mitochondrial DNA among geographically isolated grassland butterflies. J. Biogeogr. 38: 299-310.

Craft, K. J., S. U. Pauls, K. Darrow, S. E. Miller, P. D. N. Hebert, L. E. Helgen, V. Novotny V, and G. D. Weiblen. 2010. Population genetics of ecological communities with DNA barcodes: An example from New Guinea Lepidoptera. PNAS. 107: 5041–5046.

Dsouli-Aymes, N., J. Michaux, E. De Stordeur, A. Couloux, M. Veuille, and G. Duvallet. 2011. Global population structure of the stable fly (*Stomoxys calcitrans*) inferred by mitochondrial and nuclear sequence data. Infect. Genet. Evol. 11: 334–342.

Joyce, A. L., M. Sermeno-Chicas, L. Serrano-Cervantes, M. Paniagua, S. J. Scheffer, and M. A. Solis. 2016. Host-plant associated genetic divergence of two *Diatraea* spp. (Lepidoptera: Crambidae) stemborers on novel crop plants. Ecol. Evol. 6: 8632–8644.

Krosch, M. N., A. M. Baker, B. G. McKie, P. B. Mather, and P. S. Cranston. 2009. Deeply divergent mitochondrial lineages reveal patterns of local endemism in chironomids of the Australian Wet Tropics. Austral. Ecol. 34: 317-328.

Marquez, J. G., M. A. Cummings, and E. S. Krafsur. 2007. Phylogeography of stable fly (Diptera: Muscidae) estimated by diversity at ribosomal 16S and cytochrome oxidase I mitochondrial genes. J. Med. Entomol. 44: 998-1008.

Mutanen, M., A. Hausmann, P. D. N. Hebert, J. F. Landry, J. R. deWaard, and P. Huemer. 2012. Allopatry as a Gordian knot for taxonomists: patterns of DNA barcode divergence in Arctic-alpine Lepidoptera. PLoS One. 7: e47214.

Mutanen, M., S. M. Kivelä, R. A. Vos, C. Doorenweerd, S. Ratnasingham, A. Hausmann, P. Huemer, V. Dincă, E. van Nieukerken, C. Lopez-Vaamonde, R. Vila, L. Aarvik, T. Decaëns, K.A. Efetov, P. D. Hebert, A. Johnsen, O. Karsholt, M. Pentinsaari, R. Rougerie, A. Segerer, G. Tarmann, R. Zahiri, and H. C. Godfray. 2016. Species-level para- and polyphyly in DNA barcode gene trees: strong operational bias in European Lepidoptera. Syst. Biol. 65: 1024–1040.

Paynter, Q., A. H. Gourlay, P. T. Oboyski, S. V. Fowler, R. L. Hill, T. M. Withers, H. Parish, and S. Hona. 2008. Why did specificity testing fail to predict the field host-range of the gorse pod moth in New Zealand? Biol. Control. 46: 453–462.

Periasamy, M., R. Schafleitner, K. Muthukalingan, and S. Ramasamy. 2015. Phylogeographical structure in mitochondrial DNA of legume pod borer (*Maruca vitrata*) population in tropical Asia and sub-Saharan Africa. PLoS One. 10: 1-24.

Silva-Brandão, K. L., L. C. Almeida, S. S. Moraes, and F. L. Cônsoli. 2013. Using population genetic methods to identify the origin of an invasive population and to diagnose cryptic subspecies of *Telchin licus* (Lepidoptera: Castniidae). Bull. Entomol. Res. 103: 89-97.

Silva-Brandão, K. L., T. V. Santos, F. L. Cônsoli, and C. Omoto. 2015. Genetic diversity and structure of Brazilian populations of *Diatraea saccharalis* (Lepidoptera: Crambidae): implications for pest management. J. Econ. Entomol. 108: 307-316.

Telfer, A. C., M. R. Young, J. Quinn, K. Perez, C. N. Sobel, J. E. Sones, V. Levesque-Beaudin, R. Derbyshire, J. Fernandez-Triana, R. Rougerie, A. Thevanayagam, A. Boskovic, A. V. Borisenko, A. Cadel, A. Brown, A. Pages, A. H. Castillo, A. Nicolai, B. Mockford, G. Mockford, B. Bukowski, B. Wilson, B. Trojahn, C. A. Lacroix, C. Brimblecombe, C. Hay, C. Ho, C. Steinke, C. P. Warne, C. Garrido Cortes, D. Engelking, D. Wright, D. A. Lijtmaer, D. Gascoigne, D. Hernandez-Martich, D. Morningstar, D. Neumann, D. Steinke, D. DeBruin, M. DeBruin, D. Dobias, E. Sears, E. Richard, E. Damstra, E. V. Zakharov, F. Laberge, G. E. Collins, G. A. Blagoev, G. Grainge, G. Ansell, G. Meredith, I. Hogg, J. McKeown, J. Topan, J. Bracey, J. Guenther, J. Sills-Gilligan, J. Addesi, J. Persi, K. K. S. Layton, K. D'Souza, K. Dorji, K. Grundy, K. Nghidinwa, K. Ronnenberg, K. M. Lee, L. Xie, L. Lu, L. Penev, M. Gonzalez, M. E. Rosati, M. Kekkonen, M. Kuzmina, M. Iskandar, M. Mutanen, M. Fatahi, M. Pentinsaari, M. Bauman, N. Nikolova, N. V. Ivanova, N. Jones, N. Weerasuriya, N. Monkhouse, P. D. Lavinia, P. Jannetta, P. E. Hanisch, R. T. McMullin, R. Ojeda Flores, R. Mouttet, R. Vender, R. N. Labbee, R. Forsyth, R. Lauder, R. Dickson, R. Kroft, S. E. Miller, S. MacDonald, S. Panthi, S. Pedersen, S. Sobek-Swant, S. Naik, T. Lipinskaya, T. Eagalle, T. Decaëns, T. Kosuth, T. Braukmann, T. Woodcock, T. Roslin, T. Zammit, V. Campbell, V. Dinca, V. Peneva, P. D. N. Hebert, and J. R. deWaard. 2015. Biodiversity inventories in high gear: DNA barcoding facilitates a rapid biotic survey of a temperate nature reserve. Biodivers. Data J. 3: e6313.

Von Reumont, B. M., J. F. Struwe, J. Schwarzer, and B. Misof. 2012. Phylogeography of the burnet moth *Zygaena transalpina* complex: molecular and morphometric differentiation suggests glacial refugia in Southern France, Western France and micro-refugia within the Alps. J. Zool. Syst. Evol. Res. 50: 38-50.

Wei, S-J., L-J. Cao, Y-J. Gong, B-C. Shi, S. Wang, F. Zhang, X. Guo, Y-M. Wang, and X-X. Chen. 2015. Population genetic structure and approximate Bayesian computation analyses reveal the southern origin and northward dispersal of the oriental fruit moth *Grapholita molesta* (Lepidoptera: Tortricidae) in its native range. Mol. Ecol. 24: 4094–4111.

Wu, Y., J. J. Molongoski, D. F. Winograd, S. M. Bogdanowicz, A. S. Louyakis, D. R. Lance, V. C. Mastro, and R. G. Harrison. 2015. Genetic structure, admixture and invasion success in a Holarctic defoliator, the gypsy moth (*Lymantria dispar*, Lepidoptera: Erebidae). Mol. Ecol. 24: 1275–1291.
